# TCMD: A High‐Throughput and Rapid Method for Screening Antimicrobial Ingredients from Renewable Bio‐Based Resources

**DOI:** 10.1002/advs.202502156

**Published:** 2025-04-28

**Authors:** Yongdong Xu, Yueyao Wang, Yongming Chen, Yunxia Wang, Shicheng Zhang, Gang Luo, Fuhao Cui, Taisheng Du, Zhidan Liu

**Affiliations:** ^1^ Laboratory of Environment‐Enhancing Energy (E2E) Key Laboratory of Agricultural Engineering in Structure and Environment of Ministry of Agriculture and Rural Affairs College of Water Resources and Civil Engineering China Agricultural University Beijing 100083 China; ^2^ Water & Energy Technologies (WET) Lab Department of Civil and Environmental Engineering Princeton University Princeton NJ 08544 USA; ^3^ National Key Laboratory of Wheat Improvement Peking University Institute of Advanced Agricultural Sciences Shandong Laboratory of Advanced Agricultural Sciences at Weifang Weifang 261325 China; ^4^ College of Pharmaceutical Sciences Zhejiang University Hangzhou 310058 China; ^5^ Shanghai Key Laboratory of Atmospheric Particle Pollution and Prevention Department of Environmental Science and Engineering Fudan University Shanghai 200433 China; ^6^ Department of Plant Pathology and the Ministry of Agriculture Key Laboratory of Pest Monitoring and Green Management China Agricultural University Beijing 100193 China; ^7^ Center for Agricultural Water Research in China China Agricultural University Beijing 100083 China

**Keywords:** antibiotic liquids, antimicrobial compound identification, biomass, high throughout, virtual screening

## Abstract

Antibiotic resistance and pathogenic infections underscore the importance and urgency of novel control agent development. Bio‐based products represent a rich reservoir of antimicrobial agents. However, traditional strategies for screening new active compounds are time‐consuming, costly, and limited by accessible resources. Here, transcriptomic combinatorial molecular docking (TCMD), a novel method enabling fast identification of antimicrobial components in complex mixtures without requiring prior knowledge is proposed. Results show that, in eukaryotic microorganism systems, TCMD demonstrates superior performances in screening antifungal compounds within hydrothermal liquefaction aqueous. The high accuracy is confirmed by molecular dynamics simulation, antifungal experiments, and RT‐qPCR (reverse transcription real‐time quantitative polymerase chain reaction) analysis. Furthermore, TCMD exhibits cross‐system applicability, as evidenced by successful antibacterial substances screening in prokaryotic systems using plant essential oil and traditional Chinese medicine from previous studies. Compared to conventional approaches, TCMD is estimated to be 3–20 times faster and ≈10 times more cost‐effective, while maintaining high‐throughput capacity for analyzing thousands of compounds simultaneously. These demonstrate that TCMD is a rapid, precise, and flexible method for antimicrobial compound discovery, significantly accelerating the development of new antibacterial agents.

## Graphical abstract

This study develops a strategy to screen the active compounds from bio‐mixtures via combining transcriptomic profiling and molecular docking. The proposed method demonstrates 100% correction in three different biobased mixtures and is 3–20 times faster than traditional approaches. This method can be widely used for screening novel active compounds, accelerating the development of new antibacterial agents.

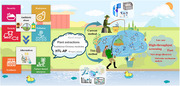

## 1 Introduction

Countless pathogenic microorganisms exist in nature, such as bacteria, fungi, viruses, oomycetes, and nematodes, posing significant threats to human and animal health, as well as food security,^[^ ^1^ ^]^ causing diseases, deaths, and crop yield reduction.^[^ ^2^, ^3^ ^]^ Antibiotic agents represent a crucial strategy for controlling pathogenic microorganisms.^[^ ^4^ ^]^ However, the increasing antibiotic usage has triggered a series of environmental and human health risks.^[^ ^5^, ^6^, ^7^ ^]^ Moreover, microbial resistance accumulation leads to reduced control efficiency.^[^ ^8^, ^9^, ^10^ ^]^ These crises necessitate the accelerated discovery of new antibiotics with environmental‐friendly and bio‐safety to ensure sustainable development.^[^ ^9^ ^]^

Plants serve as a natural reservoir of microorganism inhibitors, producing enormous metabolites with broad‐spectrum antimicrobial activity and giving reference to antibiotic development.^[^ ^11^, ^12^ ^]^ Plant extracts like traditional Chinese medicine have been widely used in human disease treatment like hypertension, diabetes, and coronary disease, and can improve the efficacy of existing antibiotics by reversing bacterial resistance.^[^ ^13^ ^]^ Similarly, biomass‐derived liquids, including biogas slurry, pyrolysis liquid, and hydrothermal liquefaction aqueous phase (HTL‐AP), are considered as potential alternatives to biosafe antibiotics.^[^ ^14^ ^]^ Specifically, HTL‐AP has been confirmed to have extensive inhibition on microorganisms including bacteria, fungi, and influenza A virus.^[^ ^15^ ^]^ It provides unlimited opportunities for new renewable antibiotic development due to unmatched chemical diversity. However, these bio‐based liquids are complex mixtures containing numerous components, complicating the qualitative and quantitative analysis of active compositions.^[^ ^12^ ^]^

Currently, screening active compounds from mixtures involves a time‐consuming and labor‐intensive procedure, including compound selection, purification (e.g., column chromatography, high performance liquid chromatography, literature comparison, model chemical testing, and elucidating the action mechanism of selected compounds.^[^ ^16^, ^17^ ^]^ These approaches face intrinsic limitations from matrix complexity and incomplete compound libraries. Though network pharmacology is widely used in screening therapeutic components of traditional Chinese medicine,^[^ ^18^, ^19^, ^20^ ^]^ its mammalian disease‐oriented databases lack microorganism‐specific target interactions, rendering it inapplicable for antimicrobial discovery.

Bioinformatics like transcriptomics has revolutionized antimicrobial research by decoding resistance mechanisms,^[^ ^21^, ^22^ ^]^ and identifying therapeutic biomarkers.^[^ ^23^, ^24^ ^]^ However, bioinformatics is currently mainly adopted to identify specific action targets of antibiotics guiding the design and development of targeted chemicals.^[^ ^25^ ^]^ The application of bioinformatics in mixture antimicrobial experiments is to reveal the inhibition pathways (the affected microbial biological functions).^[^ ^26^, ^27^ ^]^ Virtual screening, especially molecular docking, is a common technology in computer‐aided drug design, which is used to predict ligand‐target binding affinities to prioritize target proteins and bioactive chemicals.^[^ ^28^, ^29^, ^30^ ^]^ Similarly, specific compounds and proteins are also required to conduct molecular docking.

Based on the characteristics of bioinformatics and virtual screening, we hypothesized that, after treating microorganisms with mixtures, using transcriptome analysis could identify mixture biomarkers, and using molecular docking could analyze interactions between biomarkers and mixture compounds, further screening active compounds and relative proteins. Therefore, a combination of transcriptome and molecular docking can efficiently and high‐throughput identify effective antimicrobial substances in complex mixtures and their targets, without the need for existing databases or extensive experiments.

Here, we proposed a novel strategy TCMD (combination of transcriptome and molecular docking) to a) screen antimicrobial components in complex matrices and their corresponding proteins, b) construct a differential protein database in microorganisms without requiring prior knowledge. TCMDs versatility was rigorously validated across prokaryotic and eukaryotic systems using three distinct sample types (plant essential oils, traditional Chinese medicine, HTL‐AP). Additionally, general methods such as plate antimicrobial experiments of pure chemicals, molecule dynamic simulation, and reverse transcription real‐time quantitative polymerase chain reaction (RT‐qPCR) were applied to validate active substances.

## 2 Results

### 2.1 TCMD (transcriptomic combinatorial molecular docking): A Fast and High‐Throughput Method to Identify Active Constituents from Complex Mixtures with Massive Compounds

**Figure 1:**
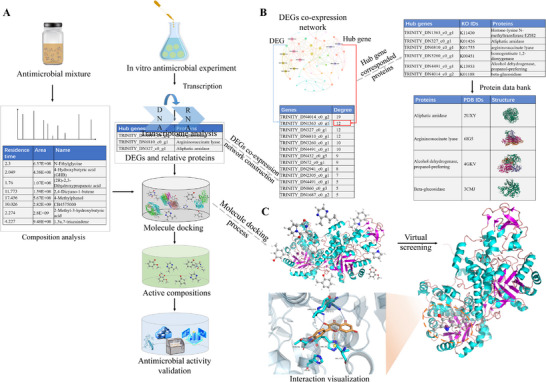
Workflow of TCMD. a) Prediction process of active compounds. b) Recognition of relative proteins. c) Procedure of antimicrobial compound screening.

The TCMD methodology synergistically integrated transcriptome analysis with molecular docking technology (**Figure** **1**a). Chemical compositions within mixtures were explicitly identified using gas chromatograohy‐mass spectrometry (GC‐MS), high performance liquid chromatography (HPLC), liquid chromatograph mass spectrometer (LC‐MS), comprehensive two‐dimensional gas chromatography–time of flight mass spectrometry (GC×GC‐TOF MS), fourier‐transform ion cyclotron resonance mass spectrometry (FT‐ICR‐MS), and other technologies, facilitating a comprehensive chemical analysis. Following the in vitro antimicrobial experiments, transcriptome analysis of whole cells was conducted on both treated and control microorganisms to get differentially expressed genes (DEGs) (The specific operation of each procedure is described in the Experimental Section and Supporting Information). Kyoto Encyclopedia of Genes and Genomes (KEGG) pathway enrichment analyses were performed on these DEGs. The DEGs within the selected KEGG pathways were then screened for hub genes via weighted gene co‐expression network analysis (WGCNA) (Figure 1b). Consequently, using the KEGG database (https://www.kegg.jp/kegg/), the relative proteins corresponding to DEGs were selected as receptors, and the constituents identified in mixtures were considered as ligands for molecular docking (Figure 1c). The components exhibiting stronger binding affinity than the defined cut‐off value were deemed as potential antimicrobial ingredients. Additionally, various methods can be applied to validate active substances, including plate experiments of pure chemicals, molecular dynamic simulation, RT‐qPCR, and so on.

### 2.2 TEST 1: TCMD for Identification of Antifungal Components from HTL‐AP

#### 2.2.1 Characterization and Antifungal Properties of HTL‐AP

**Figure 2:**
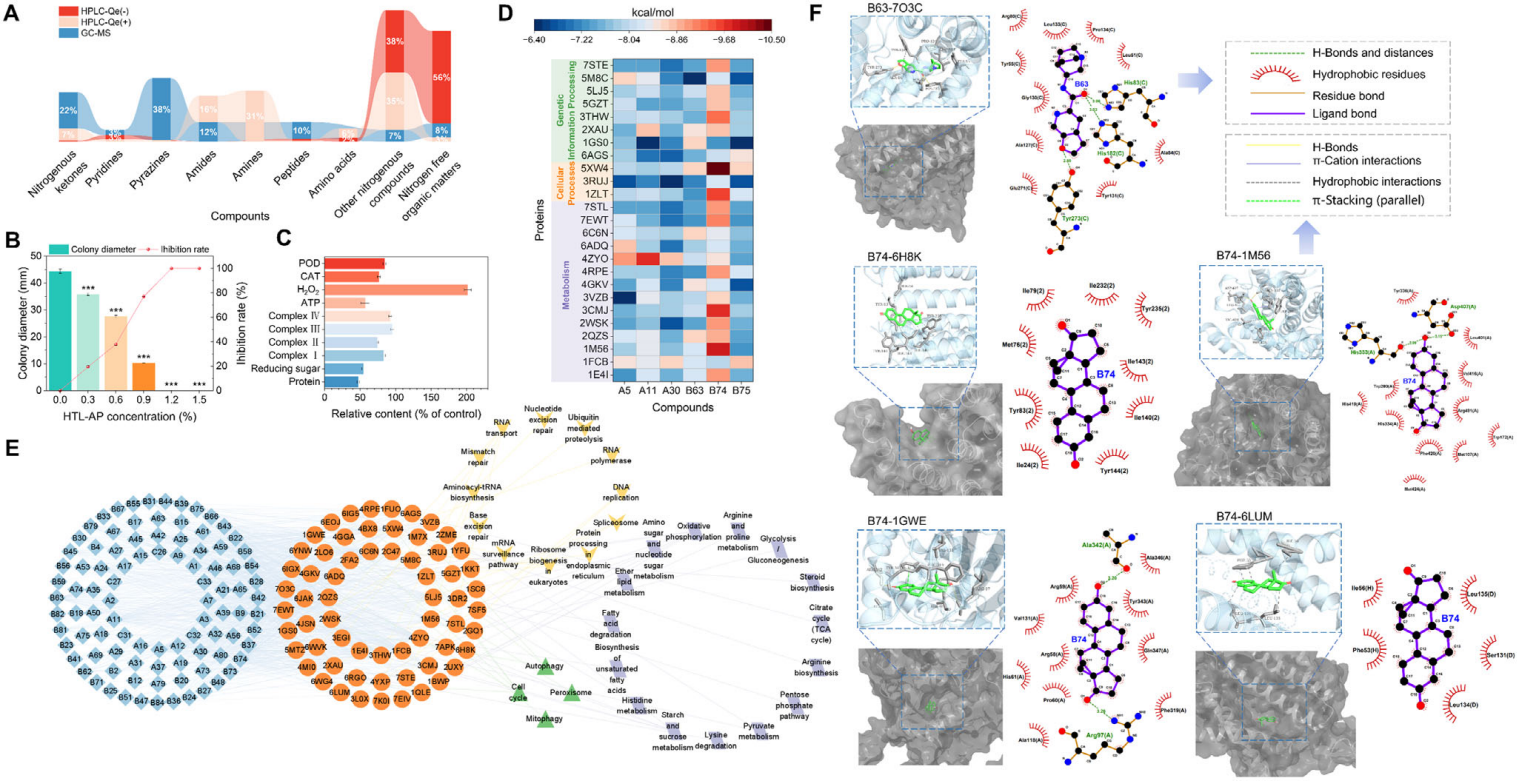
Identification of antifungal constitutions in HTL‐AP using TCMD. a) Component distribution of HTL‐AP. The specific substances are shown in Data S1 (Supporting Information). b) Inhibition rate of HTL‐APs with different concentrations. c) Effect of HTL‐AP on intracellular compound and energy metabolism. d) Docking scores of molecule docking (score <−8.5 kcal mol^−1^). The entire docking results are shown in Data S2 (Supporting Information). e) Network analysis of the relationship between potential active substances and relative proteins. f) Visualization of substance‐protein complexes, B74‐1M56 (Estrone – Mitochondrial respiratory chain complex IV, −9.9 kcal mol^−1^), B74‐1GWE (Estrone – Catalase, −10.0 kcal mol^−1^), B74‐6H8K (Estrone – Mitochondrial respiratory chain complex I, −7.5 kcal mol^−1^), B74‐6LUM (Estrone – Mitochondrial respiratory chain complex II, −8.6 kcal mol^−1^) and B63‐7O3C (PHA 543 613 – Mitochondrial respiratory chain complex III, −9.2 kcal mol^−1^). Data from antifungal experiments was presented as mean ± SEM, *n* = 6, and data from enzyme activity experiments was presented as mean ± SEM, *n* = 3. *P*‐values are calculated using one‐way ANOVA with Tukey correction, and ^*^ was used for comparisons with the control group. ^*^ *p* < 0.05, ^**^ *p* < 0.01, ^***^ *p* < 0.001.

HTL‐AP, a kind of wastewater from the energy conversion process, is considered a promising antimicrobial agent.^[^ ^14^, ^31^, ^32^, ^33^ ^]^ The physiochemical characteristics are displayed in Table S1 (Supporting Information). The major compositions (Experimental Section) of HTL‐AP were identified as nitrogenous compounds such as amide, nitrogenous ketones, pyrazines, and peptides (**Figure** **2**a).^[^ ^31^ ^]^ Substances identified by high performance liquid chromatography ‐ Q exactive (HPLC‐Qe, positive ion) were labeled as A1‐A81, those in HPLC‐Qe (negative ion) were marked as B1‐B88, and compounds identified by GC‐MS were defined as C1‐C33. *Rhizoctonia solani* (*R*. *solani*), a devastating phytopathogen causing up to 50% yield losses in staple crops threatening food supply for humans,^[^ ^34^, ^35^ ^]^ was used as a target sample. HTL‐AP demonstrated potent inhibition against *R. solani* with a MIC of 1.2% (≈11.8 mg L^−1^) (Figure 2b; Figure S1a, Supporting Information), outperforming some natural or synthetic small molecules.^[^ ^36^ ^]^ In a liquid medium, the mycelia were branched and the medium was turbid (Figure S2b, Supporting Information). Previous studies showed that metabolism impact is a major action pathway for bio‐based liquids achieving antifungal effects.^[^ ^26^, ^27^, ^37^ ^]^ ATP (adenosine triphosphate) is the most direct energy source in living organisms, and mitochondria are important sites for eukaryotic cells to produce ATP. The activity of four mitochondrial respiratory complexes could affect ATP content.^[^ ^38^ ^]^ H_2_O_2_ is a kind of reactive oxygen species toxic to cells, and CAT (Catalase) and POD (Peroxidase) are the two main enzymes removing excess H_2_O_2_ and other peroxides in cells.^[^ ^39^ ^]^ Some bio‐based matrices like plant essential oil, extracts, and antimicrobial peptides have been confirmed to decrease the activity of four mitochondrial respiratory complexes,^[^ ^40^, ^41^ ^]^ CAT and POD achieving inhibition.^[^ ^42^, ^43^, ^44^ ^]^ Therefore, these six enzymes were used to reveal HTL‐AP's influence on fungal metabolism. Results showed that HTL‐AP led to intracellular energy and compound metabolism disorder, as well as oxidative stress (CAT/POD activity reduction >60%, H_2_O_2_ accumulation 3.1‐fold), as evidenced by reduced activity of four enzymes related to energy metabolism, along with decreased levels of intracellular ATP, total sugar, and protein (activity reduction >60%) (Figure 2c).

#### 2.2.2 Receptor and Ligand Analysis

A total of 3089 DEGs were identified in *R. solani* after HTL‐AP treatment (Figure S2 and S3, Supporting Information). These DEGs were enriched into KEGG pathways, and 10 secondary pathways with >25 DEG were selected from the top three primary pathways to create gene sets respectively. Then these DEG sets were used to conduct WGCNA, and the DEGs ranked in the top 20% were regarded as hub genes (Figure S4 and Table S2, Supporting Information). Consequently, a total of 77 proteins relevant to hub genes were researched in KEGG database. Meanwhile, one of six detected enzymes (mitochondrial respiratory chain complex IV, PDB ID: 1M56) was identified via this workflow. As enzyme activity test and transcriptome analysis indicated, the other four enzymes (mitochondrial respiratory chain complex I, PDB ID: 6H8K; complex II, PDB ID: 6LUM; complex III, PDB ID: 7O3C; and Catalase, PDB ID: 1GWE) were also added into hub gene collection although they were not identified via this flow. Thereby, 81 proteins (Table S3, Supporting Information) were finally chosen as potential relative proteins for docking screening with 202 constitution ligands in HTL‐AP (Data S1, Supporting Information).

There were 94 components revealing docking score <−7 kcal mol^−1^ with some relative proteins related to the function of material and energy metabolism, cell cycle, DNA replication, and so on (Figure 2e). These findings implicated that HTL‐AP could serve as a significant source of new fungicide components capable of disrupting fungal physiological activity.

According to the enzyme activity test, five groups of chemicals and relative proteins with the strongest binding force were selected for interaction analysis (Figure 2f). B74 inserted the oxygen transfer channel of 1M56 and occupied the binding site of oxygen,^[^ ^45^ ^]^ the hydroxyl on its benzene ring formed two hydrogen bonds with residue His333 and Asp407, and its benzene ring formed a π‐C stacking interaction with residue His334. Meanwhile, B74 could also be inserted into the active site channel of 6H8K, 6LUM, and 1GWE.^[^ ^46^, ^47^, ^48^ ^]^ In 1GWE, B74's benzene ring formed parallel‐displace π–π stacking interaction with residue Tyr343, the oxygens of benzene ring and C5‐ring formed hydrogen bonds with residue Ala342 and Arg97, respectively. B63 occupied the hydrophobic channel of 7O3C and its carbonyl formed hydrogen bonds with residue His128 and His83, and oxygen on the carbon ring formed hydrogen bonds with residue Tyr273. To summarize, the components of microalgae‐derived HTL‐AP effectively inhibited oxygen metabolic activity, disordered mitochondrial respiration, and decreased ATP production,^[^ ^3^ ^]^ consistent with the laboratory results.

Additionally, some chemicals and their relative proteins that had the strongest binding force were extracted to investigate their binding modes (Figure S5, Supporting Information). Visualization analysis revealed that active ingredients of HTL‐AP could be inserted into the active site channels of relative proteins, forming hydrophobic interaction, π–π stacking interaction, π‐cation interaction, salt bridge, and hydrogen bond, thereby disrupting protein functions.

#### 2.2.3 Validation of Active Components: Molecular Dynamic Simulation

**Figure 3:**
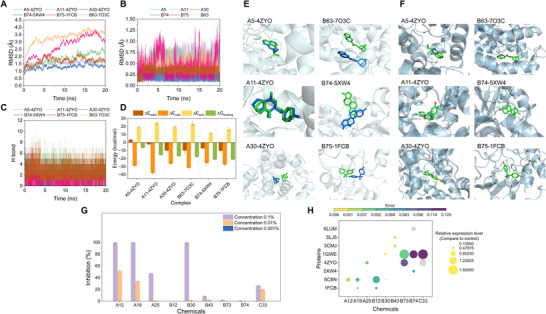
Validation of active components identified from HTL‐AP via TCMD. A5‐4ZYO (2‐ethyl‐1H‐perimidine – Stearoyl‐CoA desaturase, −9.1 kcal mol^−1^), A11‐4ZYO (3‐[(4‐hydroxyphenyl)methyl]‐octahydropyrrolo[1,2‐a]pyrazine‐1,4‐dione – Stearoyl‐CoA desaturase, −9.8 kcal mol^−1^), A30‐4ZYO (Harmaline – Stearoyl‐CoA desaturase, −8.8 kcal mol^−1^), B74‐5XW4 (Estrone – Tyrosine‐protein phosphatase, −10.5 kcal mol^−1^), B75‐1FCB (Bisphenol A – L‐lactate dehydrogenase, −8.8 kcal mol^−1^) and B63‐7O3C (PHA 543 613 – Mitochondrial respiratory chain complex III, −9.2 kcal mol^−1^). a) RMSD of complexes during MDS. b) RMSD of chemical ligands during MDS. c) HBs between relative proteins and chemical ligands in complexes during MDS. d) Bending free energy between relative proteins and chemical ligands after MDS. e) Changes in ligand conformation before and after MDS. f) Interactions between proteins and chemical ligands after MDS. g) Chemicals and antifungal capacity h) RT‐qPCR analysis of relative proteins. Data from antifungal experiments were presented as mean ± SEM, *n* = 6, and data from RT‐qPCR experiments were presented as mean ± SEM, *n* = 3.

Molecular dynamic simulation (MDS) was employed to assess the stability and evolution of the docking conformations of chemical‐protein complexes in the CHARMM force field.^[^ ^30^, ^49^ ^]^ We adopted a cutoff value of −8.5 kcal mol^−1^ to filter docking results,^[^ ^50^ ^]^ and six complexes were used to conduct MDS. Root mean square deviation (RMSD) analysis (Experimental Section) revealed system equilibration within 5 ns for five complexes, while B75‐1FCB required extended relaxation (**Figure** **3**a). Meanwhile, the RMSD of complexes B74‐5XW4, A30‐4ZYO, A11‐4ZYO, and A5‐4ZYO remained below 2.5 Å throughout the entire simulation, defining their better structural integrity.^[^ ^51^ ^]^ Similarly, almost all chemical ligands whose RMSD slightly fluctuated under 0.75 Ǻ had good stability (Figure 3b). After MDS, chemical ligand conformations slightly vibrated or shifted in the active site, which might owe to flexible relative proteins. The number of hydrogen bonds (HB) formed between components and relative proteins during the MDS process showed that chemicals A11, A30, and B74 could form very stable H bonds with relative proteins (Figure 3c; Figure Table S4, Supporting Information), consistent with the RMSD results.

The MM‐GBSA bending free energy (ΔG_binding_) calculations ranked complexes as: B75‐1FCB (−21.25 ± 0.04 kcal mol^−1^), B74‐5XW4 (−21.15 ± 0.03 kcal mol^−1^), A30‐4ZYO (−18.18 ± 0.05 kcal mol^−1^), B63‐7O3C (−17.80 ± 0.05 kcal mol^−1^), A11‐4ZYO (−15.47 ± 0.04 kcal mol^−1^) and A5‐4ZYO (−6.65 ± 0.04 kcal mol^−1^) (Figure 3d). G_solv_ (Solvation energy) of compounds can impair ΔG_binding_, and changes in compound binding state caused by the movement of proteins during MDS will impact ΔG_binding_, resulting in differences between ΔG_binding_ ranking and molecular docking results.^[^ ^28^ ^]^ The above results symbolized that the docking results were relatively reasonable.

#### 2.2.4 Validation of Active Components: Plate Antifungal Experiment

Based on TCMD results, compounds were categorized into four groups, and nine compounds were randomly selected for antifungal evaluation via colony growth assays. The results indicated that all chemicals demonstrated various influences on fungi growth (Figure 3g; Figure S6, Supporting Information), especially, A12 (4‐Aminobiphenyl), A19 (6‐Methylquinoline), A25 (Benzidine), and C33 (Dibutyl phthalate) had an observable antifungal effect with inhibition rate ranging from 26.7% to 100% at concentration 0.1 %. Noticeably, B12 (9(Z),11(E)‐Conjugated linoleic acid) significantly changed mycelial traits.

#### 2.2.5 Validation of Active Components: Identification in the RNA Expression Level

Based on plate experiments and molecular docking results, eight significantly regulated relative proteins after HTL‐AP treatment were selected and then utilized to conduct RT‐qPCR analysis (Table S5, Supporting Information). Their relative gene expression levels in *R*. *solani* treated with the selected nine chemicals relative to control groups were assessed, respectively (Figure 3h). The results indicated that the 9 compounds significantly affected the expression of genes with higher binding affinities, as predicted by molecular docking results. Therefore, we concluded that the RT‐qPCR results validated the accuracy of the TCMD method.

In summary, TCMD enabled the rapid identification of 81 novel antimicrobial candidates from a complex mixture. More importantly, most of them were not considered antimicrobial candidates before, expanding the current knowledge of antimicrobial compounds and action relative protein, and facilitating novel agent and action target discovery. Many other mixtures possess similar properties and antimicrobial potential to HTL‐AP, like plant extracts, pyrolysis liquid, and biogas slurry, illustrating the broad application of the TCMD approach.

### 2.3 TEST 2: TCMD for Antibacterial Components from Essential Oil and Traditional Chinese Medicine

To validate TCMD's applicability, two complex mixtures, essential oil (EO) from *Cinnamomum camphora* (*C*. *camphora*) and *Ginkgo biloba exocarp* extract (GBE) from traditional Chinese medicine *Ginkgo biloba exocarp*, were used as samples for active compound identification, both of which have previously demonstrated antimicrobial potential. EO and plant extracts are elaborate mixtures of plant‐derived volatile compounds,^[^ ^52^ ^]^ and exhibit broad‐spectrum bioactivities including antibacterial, antiviral, antifungal, and insecticidal activities, which was reckoned as an antibiotic candidate.^[^ ^53^ ^]^ However, the massive compounds in EO and GBE pose a challenge for active compound identification. To date, EO compounds like caryophyllene oxide, β‐caryophyllene, spathulenol, germacrene D, and heptacosane have been identified as antimicrobial compounds,^[^ ^54^ ^]^ while GBE compounds, such as ginkgolide, bilobalide, ginkgolic acid, isoginkgetin, and sciadopitysin have been identified to have antimicrobial activity.^[^ ^55^ ^]^

Studies have investigated the impact of EO on *Escherichia coli* (*E. coli*) transcriptome and the effect of GBE on *Staphylococcus haemolyticus* (*S*. *haemolyticus*) transcriptome.^[^ ^56^, ^57^ ^]^ We applied TCMD to explore antibacterial components in EO and GBE based on the published data resources.

**Figure 4:**
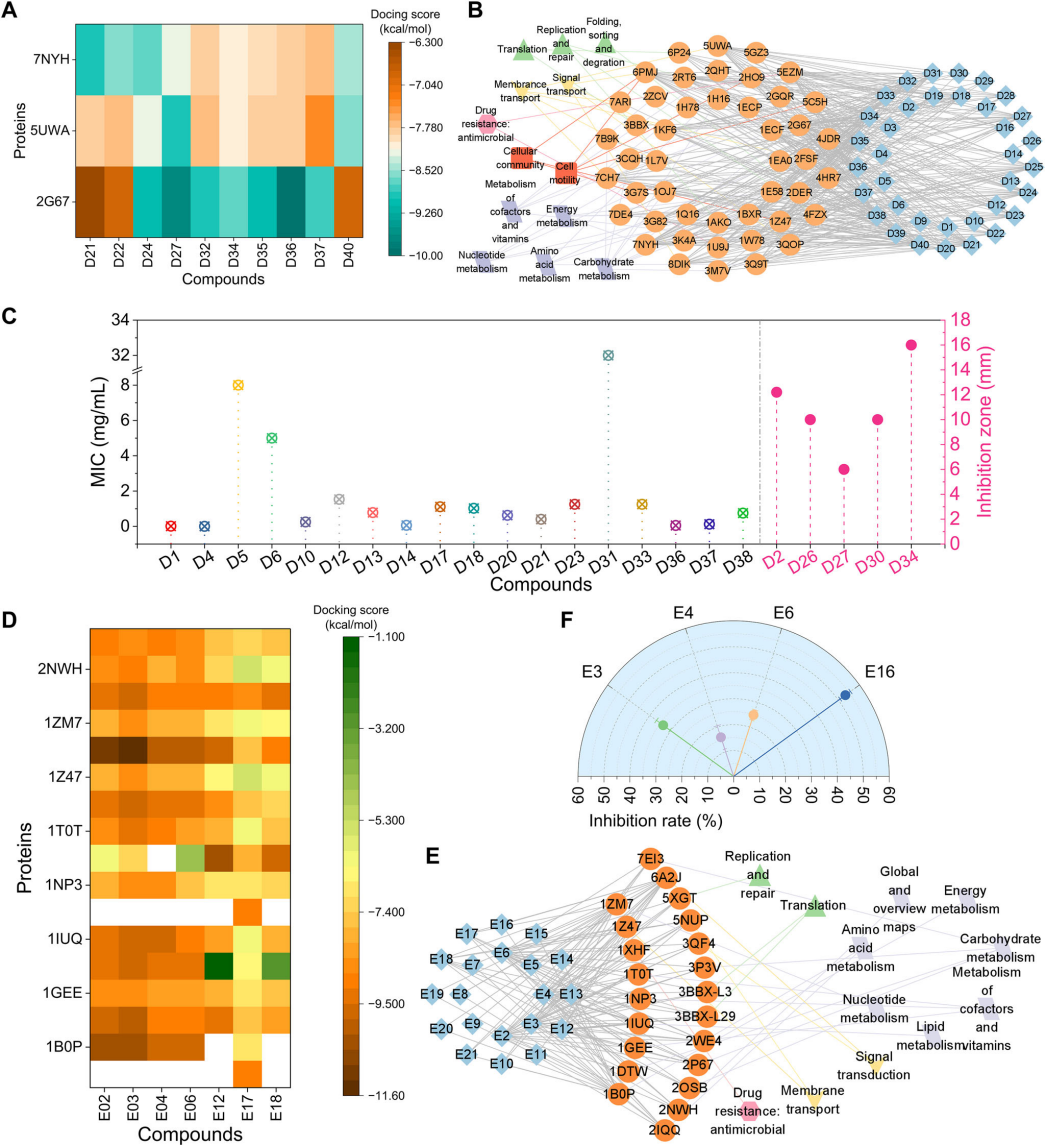
Identification of antibacterial ingredients in EO and GBE via TCMD. a) Results of molecule docking (score <−8.5 kcal mol^−1^). The entire docking results are shown in Data S3 (Supporting Information). b) Network of potential active substances and relative proteins. c) Inhibition effect of screened components on *E*. *coli* in previous and this study. d) Results of molecule docking (score <−8.5 kcal mol^−1^). The entire docking results are shown in Data S4 (Supporting Information). e) Network of potential active substances and relative proteins. f) Inhibition effect of selected 4 components on *S*. *haemolyticus*. Data of antibacterial experiments presented as mean ± SEM, *n* = 3.

In terms of the antibacterial activity of EO on *E*. *coli*, we analyzed the dataset (GSE160284) and constructed WGCNA to refine hub genes. Then 118 corresponding proteins of hub genes were gained (Table S6, Supporting Information) and 40 constituents of *C*. *camphora* oil were acquired from a previous study (Table S7, Supporting Information) ^[^ ^58^ ^]^ to conduct TCMD to screen antibacterial components (Data S3, Supporting Information). Totally 36 compounds were identified as potential active antimicrobial compounds owing to their strong binding affinities with some relative proteins, with docking scores below −7 kcal mol^−1^ (**Figure** **4**b).^[^ ^59^ ^]^ Ingredients D21, D22, D24, D27, D32, D34, D35, D36, D37, and D40, with docking scores below −8.5 kcal mol^−1^, (Figure 4a), might be ingredients with strong antibacterial activity. Some identified active compounds have been ascertained to have an inhibition effect on *E*. *coil* with MIC of 0.001‐32.0 mg/mL (Figure 4c).^[^ ^60^, ^61^, ^62^, ^63^, ^64^, ^65^, ^66^, ^67^, ^68^, ^69^, ^70^, ^71^, ^72^ ^]^ Through traditional methods, identification of these compounds would cost massive work (about several months). Some ingredients have also been confirmed to inhibit other bacteria, fungi, and insects (Table S13, Supporting Information). Interestingly, we also identified some compositions, whose antimicrobial activity has not been previously reported, but have bioactivities such as anti‐inflammatory and anticancer effects.^[^ ^73^, ^74^ ^]^ Among these compounds, chemical D2 (β‐Phellandrene) was randomly chosen to test inhibition against *E*. *coil*, which demonstrated an inhibition zone of 12.2 ± 0.01 mm at a concentration of 50 mg mL^−1^. As for the 4 compounds that have not been screened for *E*. *coil* inhibitory activity, we randomly selected three substances for plate testing and found none of them had an inhibition effect on *E*. *coil*.

As for the inhibition effect of GBE on *S*. *haemolyticus*, we analyzed the downloaded transcriptome data (BioProject PRJNA850952) and constructed WGCNA to refine hub genes. Then 25 corresponding proteins of hub genes were gained (Table S8, Supporting Information) and 20 constituents of *Ginkgo biloba* L. fruit extract were acquired from a previous study (Table S9, Supporting Information) to conduct TCMD to screen antibacterial components (Data S4, Supporting Information). Totally 20 compounds were identified as potential active antimicrobial compounds owing to their strong binding affinities with some relative proteins whose docking score was <−7 kcal mol^−1^ (Figure 4e). Substances E2, E3, E4, E6, E12, E17, and E18, with docking score below −8.5 kcal mol^−1^ (Figure 4d) might be ingredients with strong antibacterial activity. Furthermore, chemicals E3, E4, E6, and E16 were randomly opted to test the antibacterial effect on *S*. *haemolyticus*, which revealed inhibition rates of 15.8–53.3% with the concentration of 0.2 mg mL^−1^ (Figure 4f). Surprisingly, chemical E6 (Sciadopitysin) has never been reported to have antibacterial ability. What's more, previous studies have ascertained some identified active substances displayed an inhibition effect on *Staphylococcus aureus* or owned pharmacological activity (Table S14, Supporting Information).

Therefore, TCMD enabled the precise discovery of new antibacterial active components in *C*. *camphora* EO and GBE, achieving rapid high‐throughput screening. Meanwhile, their molecular action mechanism could also be visualized.

## 3 Discussion

Developing antibiotic alternatives that are environmentally friendliness, low antibiotic resistance, and biosafety is important for safeguarding public health, advancing sustainable agriculture, ensuring global food security, and preserving the ecological environment. Identification of active components from chemical mixture is an important prerequisite for the preparation of new antibiotic agents, which can ensure a high inhibitory intensity and maintain a stable effect of agents. However, current approaches are mainly based on literature comparison to predict active components and need model chemicals for validation making it inefficient and time‐consuming.

Here we developed an approach, TCMD, based on bioinformatics and molecular docking technology. We precisely trace relative proteins according to DEGs gained from transcriptome analysis. By molecular docking of mixture components with relative proteins, we identified potential antimicrobial compositions, achieving a verification accuracy rate of ≈100%. The comparison of TCMD and traditional methods was conducted according to our practical experimental experiences (Table S15, Supporting Information). For a mixture containing 100 compounds, TCMD would take ≈1–2 days to screen potential antimicrobial compounds, while traditional methods would require over 10 days. Meanwhile, TCMD would cost ≈550 $, while traditional methods would cost 4000 to 5500 $. Hence, TCMD overcomes the limitations of traditional methods, providing a rapid, accurate, and broadly applicable method for the identification of active substances in biogenic antimicrobial mixtures (**Table** **1**).

**Table 1:** The comparison of traditional and TCMD methods for antimicrobial compounds from massive compounds.

Indicators	Traditional method	TCMD method
Screening basis	Existing cognition	Binding force
Core technology	Literature review + Experiment	Transcriptome + molecular docking
Sample size	Limited	Countless samples theoretically
Screening time	Day‐Month	h‐day
limited to previous research	Yes	No
Co‐benefits of screening new drugs	No	Yes
Co‐benefits of screening new targets	No	Yes
Co‐benefits of molecular mechanism analysis	No	Yes
Cost	>> ten thousand $ dollars	< a thousand $ dollars
Accuracy	Very High	High

Although TCMD showed high accuracy in predicting active compounds, its performance could be further enhanced by refining hub gene selection via DEG enrichment and WGCNA, as well as clarifying the functions of enriched DEGs. In TCMD, the relative proteins were selected based on hub gene clarification via DEG enrichment and WGCNA. Regarding the identification of DEGs, transcriptome analysis is an efficient technology for gene expression analysis,^[^ ^23^ ^]^ but the previous study showed that the difference in library preparation and sequencing, and the choice of normalization procedure would affect DEG detection.^[^ ^75^ ^]^ In Test 1, four of five tested enzymes were not selected as relative proteins via WGGNA, although their activity declined. The genes related to mitochondrial respiratory chain complex II were even not considered as DEGs. Increasing samples may improve the accuracy of hub gene and relative protein as WGCNA is advised to conduct for >15 samples, while all three tests here were based on 6 samples. Meanwhile, concerning the DEG enrichment, its method is constructed by significance analysis of gene expression differences, which may be unsuitable by *p*‐value comparison.^[^ ^76^ ^]^ Besides, the screening methods and rules of hub genes may also induce the same importance of every DEG in WGCNA calculation. Previous studies showed that different enriched databases led to various results and had their limitations, some annotations might be outdated or lack experimental validation,^[^ ^23^, ^77^ ^]^ and the choice of pathway analysis methods also led to bias in hub gene identification.^[^ ^78^ ^]^ Hence, the other factor improving the accuracy is making more clear about the function of enriched DEGs. Thereby, we can speculate whether the corresponding binding chemicals inhibit or promote the growth of microorganisms, or has almost no effect, based on the role of the relative protein. Besides, TCMD was based on two assumptions: 1) compositions in mixtures were characterized as comprehensively as possible; 2) all compounds could penetrate or destroy cell membranes to interact with the protein system.

Despite these improvement strategies, TCMD has successfully screened antimicrobial components within complex mixtures with accuracy. Molecular dynamic simulation and traditional molecular biological experiment was applied to proclaim the correctness of TCMD results. As its performance in active antimicrobial compound identification, we can address that TCMD could be used for antifungal and antibacterial (including gram‐positive and negative species) component identification from mixtures.

## 4 Conclusion

In the present study, we developed a new strategy TCMD based on transcriptome and molecular docking, enabling the screening of antimicrobial components in complex mixtures and relative proteins in indicator organisms. We have demonstrated, in both prokaryotic and eukaryotic microorganism systems, the broad applicability of TCMD for identifying active substances in biogenic antimicrobial mixtures, like traditional Chinese medicine, HTL‐AP, and plant extracts, suggesting that TCMD could also be suitable for other similar mixtures like pyrolysis liquid and biogas slurry. Traditional strategy for antimicrobial compound screening is a time and cost‐consuming process including several steps, i.e., literature review, separation and purification, potential active compounds selection, and antimicrobial test. Our current research focuses on overcoming these limitations by developing a new screening method. Conducting bioinformatics analysis could construct a potential target protein database for microorganisms. Using molecular docking could fast and high‐throughput identify antimicrobial components in complex mixtures and gain a pool of interactions between compounds and proteins without requiring prior knowledge. TCMD is an accurate (∼100%), faster (3∼20 times), and cheaper (10 times less) method than traditional approaches. We believe that further studies involving the comprehensive detection technology of compounds and the identification of specific hug genes are key factors in achieving more accurate compound screening.

## 5 Experimental Section

### HTL‐AP and Its Antifungal Property Assessment

Microalgae *Spirulina sp* biomass was purchased from Xindaze Co., Ltd (Fujian, China) and used as the feedstock of HTL and the biochemical analysis was shown in Table S1 (Supporting Information). The HTL‐AP was obtained according to our previous report (Figure S7, Supporting Information).^[^ ^32^, ^79^ ^]^ The feedstock at 14 wt% (dry base) was added into a Parr reactor (Parr 4848, USA) and nitrogen was purged at 0.5 MPa. The HTL condition was set at 320 °C for 30 min and then HTL‐AP was collected. The pH and conductivity of HTL‐AP were measured by a pH Meter (FE28, METTLER TOLEDO, China) and Conductivity Meter (FE30, METTLER TOLEDO, China).^[^ ^32^ ^]^ The chemical oxygen demand, NH_4_‐N, and total N were measured according to the previous report^[^ ^80^ ^]^ (Table S1, Supporting Information).

GC‐MS (Agilent 7890A, Agilent Technologies, USA)^[^ ^81^ ^]^ and HPLC‐Qe HRMS (U3000‐Q Exactive, Thermo Fisher) were used to identify the chemical compounds in HTL‐AP. The GC‐MS analysis was performed according to previous reports.^[^ ^32^ ^]^ The detailed GC‐MS information is listed in Table S10 (Supporting Information). The HPLC‐Qe analysis was performed on an Ultimate 3000 UHPLC (Thermo Scientific, Massachusetts, USA) equipped with a Q Exactive Mass Spectrometer (Thermo Scientific, Massachusetts, USA). The detailed HPLC‐Qe information is listed in Table S11 (Supporting Information).

*R*. *solani* (CGMCC 3.17046) was purchased from China General Microbiological Culture Collection Center (CGMCC) and cultured by potato dextrose agar plates (PDA, 200 g potato, 20 g glucose, 15 g agar powder, and 1000 ml deionized water). The antifungal intensity of HTL‐AP was determined by the colony growth inhibition index according to previous studies.^[^ ^82^ ^]^ HTL‐AP was added into PDA to prepare plates containing 0%, 0.3%, 0.6%, 0.9%, 1.2%, and 1.5% HTL‐AP respectively. The experiments were repeated six times. The diameter of the colony was measured and hyphae morphology on plates was observed with a biomicroscope (BX51, OLYMPUS, Japan) after incubation of 72 h at 28 °C. The inhibition rate on hyphae growth was calculated by the following equation:

(1) $$I\%=\frac{C-T}{C-d}\times 100\%$$

where C is the colony diameter of plates without HTL‐AP, d is the diameter of the mycelial inoculum (5 mm), T is the diameter of plates with different HTL‐AP concentrations, and I is the inhibition index (%).

Six 5‐mm fungal mycelia inoculums of *R. solani* were added into 100 mL Potato dextrose broth (PDA without agar powder) medium with 0 or 0.3% HTL‐AP, respectively. Then it was incubated at 28 °C and 180 rpm for 72 h. Then the intracellular contents of ATP and H_2_O_2_, the content of protein and total reducing sugar, and the activities of four mitochondrial respiratory chain complexes (Complexes I, II, III, and IV), Catalase (CAT) and Peroxidase (POD) in hyphae were measured by commercial assay kits (Solarbio, China) followed by operation instructions (Procedure S1–S10, Supporting Information), respectively.^[^ ^83^, ^84^, ^85^ ^]^ The experiments were repeated three times.

### Antibacterial Component Identification from EO and GBE via TCMD

We download transcriptome sequencing of RNA from *E*. *coli* treated with EO (NCBI Series GSE160284) ^[^ ^56^ ^]^ and from *S*. *haemolyticus* treated with GBE (NCBI BioProject PRJNA850952) to analyze DEGs with the online platform of the Majorbio Cloud Platform. The hub genes were extracted by WGCNA and the differential proteins were obtained from the KEGG database. The constituents in EO and GBE were gained from previous studies.^[^ ^57^, ^58^ ^]^ Then differential proteins and constituents were used to conduct TCMD to screen antibacterial components.

### Transcriptome Analysis of Fungus

The total RNA of the mycelia treated with/without HTL‐AP was collected to conduct reverse transcription for the complementary DNA library. The procedure and methods were according to previous reports.^[^ ^86^, ^87^ ^]^ The expression of each gene in the HTL‐AP treatment group was compared with that in the control group. To identify DEGs (differential expression genes) between two different samples, the expression level of each transcript was calculated according to the transcripts per million reads (TPM) method. RSEM software was used to quantify gene abundances.^[^ ^88^ ^]^ Essentially, differential expression analysis was performed using the DESeq2. DEGs with |log2 Fold Change (FC)| ≥ 1 and FDR≤0.05 were significantly different expressed genes. In addition, functional enrichment analysis was performed to identify which DEGs were significantly enriched in KEGG pathways. The data was analyzed on the online platform of the Majorbio Cloud Platform with three biological replicates.^[^ ^87^ ^]^ The hub genes and relative proteins were selected via weighted gene co‐expression network analysis (WGCNA).^[^ ^86^ ^]^

### Active Compounds Identification via Molecular Docking

The 3D structures of components detected in HTL‐AP, EO, and GBE were obtained from NCBI PubChem (https://pubchem.ncbi.nlm.nih.gov/). The 3D structures of relative proteins and nucleic acids were acquired from Protein Data Bank (https://www.pdbus.org/).^[^ ^89^ ^]^ The retrieved structures were displayed in Tables S3, S6, and S8 (Supporting Information). For reverse docking, proteins were pretreated by AutoDock Tools, including water removal, and hydrogen addition. The ligands were also prepared in AutoDock Tools, such as the addition of hydrogen, gasteiger charges, detecting root, and choosing torsions. Then the GetBox Plugin in PyMOL software was used to predict active pockets of proteins. Finally, Auto Dock Vina was used to implement reverse docking simulation.

### Molecular Dynamic Simulation for Molecular Docking Rationality Evaluation

To further evaluate the rationality of docking conformational space, the best binding pose from a complex system was extracted for the molecular dynamic simulation process. Ligand parameters were generated for Charmm force filed by the CHARMM‐GUI web server (https://charmm‐gui.org/). Protein parameters were generated for the same force filed by VMD. VMD was also used to create a water box as a solvation and electro‐neutralize the system.^[^ ^90^ ^]^ Subsequently, molecular dynamic simulation was executed using the NAMD package.^[^ ^91^ ^]^ First, energy minimization was performed to remove steric clashes and optimize of structure.^[^ ^92^ ^]^ In the first step of 1 picosecond of NVT equilibration, the system was heated up to 300 K to stabilize the temperature of the system.^[^ ^93^ ^]^ A constraint of the backbone was applied using the NPT ensemble (P = 1 atm, T = 300 K), and the long‐range electrostatic interactions were considered by the Particle Mesh Ewald (PME) method.^[^ ^94^ ^]^ The integration time step was set to 2.0 fs and the trajectory coordinates were dumped every 500 time steps.^[^ ^95^ ^]^ The hole simulation lasted for 20 ns using CHARMM force field,^[^ ^30^, ^94^ ^]^ all parameter files were displayed in Software S1 (Supporting Information). The VMD was also used to analyze the simulation trajectories.^[^ ^90^ ^]^

Binding free energy calculation: We extracted 4000 conformations from the last 2 ns MDS trajectories in the equilibrium phase by VMD and further used MolAICal to calculate the binding free energy of small molecule‐protein complex (ΔG_binding_) based on MM‐GBSA.^[^ ^96^, ^97^, ^98^ ^]^ The calculation command is listed in Table S12 (Supporting Information) and specific scripts are shown in Software S2 (Supporting Information).

### Traditional Molecular Biological Verification

Nine compounds (A12 CAS 92‐67‐1, A19 CAS 91‐62‐3, A25 CAS 92‐87‐5, B12 CAS 60‐33‐3, B30 CAS 133‐32‐4, B42 CAS 2507‐55‐3, B73 CAS 709‐19‐3, B74 CAS 53‐16‐7, C33 CAS 84‐74‐2) were randomly selected from potential active compounds in HTL‐AP and bought from MACKLIN (Shanghai, China) for validation test via traditional molecular biological methods. Based on quantitative analysis results of HTL‐AP compositions in our other experiments, the concentrations of substances typically range from 0.001% to 0.1%. Hence, we decided to test the antifungal ability of these compounds at three different concentrations: 0.1%, 0.01%, and 0.001%. The selected compounds were dissolved in dimethyl sulfoxide (0.4% DMSO, v/v) and then added to the PDA and PDB medium to obtain a series of solutions with different concentrations (0.1%, 0.01%, and 0.001%). The PDA and PDB mediums with 0.4% DMSO (v/v) were used as a blank control. The experiments were repeated three times.

The plates were used to culture *R. solani* to evaluate the growth inhibition with Eq. (1). And then mRNA in liquid medium (0.01%) hyphae was extracted to conduct quantitative reverse transcription PCR (RT‐qPCR) analysis for fold change calculation via the 2^−ΔΔCT^ method, respectively.^[^ ^99^ ^]^ The relative expression levels of the selected genes in the experimental group relative to the control group were further analyzed. The relative genes and premiers are shown in Table S5 (Supporting Information). Three inactive compounds (D8 CAS 78‐70‐6, D11 CAS 507‐70‐0, D15 CAS 76‐49‐3) and one screened active chemical D2 (CAS 555‐10‐2) in EO, purchased from Macklin Biochemical Technology (Shanghai, China), were test against gram‐negative bacteria *Escherichia coli* (DH 5ɑ) via agar diffusion method. The selected compounds were dissolved in dimethyl sulfoxide (0.4% DMSO, v/v) and a series of solutions with different concentrations were obtained (1%, 0.5%, 0.1%, 0.05%, 0.01%, and 0.005% for D8, D11, D15, and 10%, 5% for D2). LB medium of *E*. *coli* with 5 mm diameter wells were prepared and 60 µL chemical solutions were added in wells. 0.4% DMSO (v/v) was added to well as a blank control and streptomycin was used as a positive control. Then, the plates were incubated for 24 h at 37 ± 2 °C. The inhibition zone (mm) around the well was measured to record the antibacterial activity of substances. The experiments were repeated three times.

Four potential active substances (E3 CAS 1617‐53‐4, E4 CAS 548‐19‐6, E6 CAS 521‐34‐6, E16 CAS 16611‐84‐0) were purchased from Macklin Biochemical Technology (Shanghai, China) to test antibacterial ability against *Staphylococcus haemolyticus* (ATCC 29 970). All chemicals were dissolved in DMSO at a concentration of 2.0 mg mL^−1^ and 20 µL of each solution was poured into 96‐well microplates which contained 180 µL bacterial suspension to achieve a final concentration of 0.2 mg mL^−1^ according to the previous study.^[^ ^100^ ^]^ Then the plates were incubated at 37 ± 2 °C for 16 h and the OD630 of supernatants was detected to calculate the inhibition rate. The experiments were repeated three times. The inhibition rate was calculated by the following equation:

(2) $$I\%\;=\frac{OD_{c}-OD_{T}}{OD_{C}}\times 100\%$$

where OD_c_ is the OD_630_ of plates without chemicals, OD_T_ is the OD_630_ of plates with chemicals, and I is the inhibition index (%).

### Statistical Analysis

The original data were used to calculate means and standard errors of the means (SEM). The data were presented as mean ± SEM in this article. The sample size for antifungal and antibacterial experiments was 6, and the sample sizes for enzyme activity and antibacterial experiments were 3. One‐way analysis of variance (ANOVA) followed by a Tukey post‐hoc test was carried out for statistical analysis. In all cases, significance was defined as p < 0.05 The data were sorted and calculated using Excel 2021, and the Origin 2024 software (OriginLab, Northampton, MA, USA) was used to conduct statistical analysis.

## Conflict of Interest

The authors declare no conflict of interest.

## Author Contributions

Y.X. and Y.W. contributed equally to this work. Y.X. and Y.W. performed conceptualization, data curation, formal analysis, writing – original draft, investigation, writing – review & editing in the original and revised version. Y.C. and Y.W. performed data curation, formal analysis, writing – review & editing of the original draft. S.Z. performed writing – review & editing of the antifungal experiment in the original draft. G.L. performed writing – review & editing of the antifungal experiment in the original draft. F.C. performed writing – review & editing of genetic analysis in the original draft. T.D. performed writing – review & editing of discussion in the original draft, co‐supervision. Z.L. performed conceptualization, data curation, formal analysis, funding acquisition, project administration, supervision, writing – review & editing in the original and revised versions.

## Supporting information



Supporting Information

## Data Availability

The data that support the findings of this study are available from the corresponding author upon reasonable request.
