# Supplementary material for: TCMD: A High‐Throughput and Rapid Method for Screening Antimicrobial Ingredients from Renewable Bio‐Based Resources
Source: Adv Sci (Weinh). 2025 Apr 28;12(27):2502156. doi: 10.1002/advs.202502156 (PMC12279177; doi:10.1002/advs.202502156)
Supplement: Supplementary file 1 — Supporting Information [file ADVS-12-2502156-s001.docx]

Supporting Information

TCMD: A precise, high-throughput and rapid method for screening antimicrobial ingredients from renewable bio-based resources

Yongdong Xu^#^, Yueyao Wang^#^, Yongming Chen, Yunxia Wang, Shicheng Zhang, Gang Luo, Fuhao Cui, Taisheng Du, Zhidan Liu*

# These two authors contribute equally to this work.

*Corresponding author: Dr. Zhidan Liu

Tel.: +86-10-62735568

E-mail address: zdliu@cau.edu.cn

**This file includes:**

Supplementary Text

Figures S1 to S7

Tables S1 to S15

Data S1 to S7

Software S1 to S2

References (1 to 13)

**Other Supplementary Materials for this manuscript include the following:**

Data S1 to S7

Software S1 to S2

1. Supplementary Text

Procedures for detection of intracellular compounds and energy metabolism content

1.1. Procedure S1: Hydrogen Peroxide (H2O2) content

Sample extraction: Collect 0.1 g sample to centrifuge, discard the supernatant, suggested 5 million with 1mL of regent I, splitting bacteria and cell with ultrasonication (power 20%, work time 3 s, interval 10 s, for 30 times), centrifuge at 8000 g and 4℃ for 10 min, supernatant is placed on ice for test.

Determination procedure:

1. Preheat spectrophotometer/microplate reader for 30min, adjust wavelength to 415 nm, and set zero with distilled water.

2. Incubate Solution Ⅱ, Ⅲ and Ⅳ at 37℃ (mammals) or 25℃ (other animals) water bath for more than 10min.

3. Standard working solution: If using a 96-well plate, dilute the 1mmol/mL standard solution to 2 μmol/mL standard solution with acetone, and use a trace glass colorimetric method to dilute 1mmol/mL standard solution to 1 μmol/mL standard solution.

4. Add reagents with the following list (reaction in EP tube):

**Supplementary text Table 1.**

| Reagent (μL) | Test Tube (AT) | Standard Tube (AS) | Control Tube (AC) |
| --- | --- | --- | --- |
| sample | 250 |  |  |
| Standard working solution |  | 250 |  |
| Reagent Ⅰ |  |  |  |
| Reagent Ⅱ | 25 | 25 |  |
| Reagent Ⅲ | 50 | 50 |  |
| 4000 g, room temperature centrifuge for 10 min, discard supernatant | | | |
| Reagent Ⅳ | 250 |  |  |

Add Regent Ⅳ to dissolve the precipitate (the step can remove the vegetable pigment with acetone 3-5 times), and place it at room temperature for 5 min, transfer 200 μL to a micro glass cuvette or 96-well plate and measure the absorbance at 415 nm. The control tube needs only be tested once or twice. Calculate ∆A_T_ =A_T_-A_C_, ∆A_S_=A_S_-A_C_.

Calculation:

$$H_{2}O_{2} \left( \mu mol/g \right)=\Delta A_{T}\div\left( \Delta A_{S}\div C \right)\times V1\div\left( V_{S}\div V_{E}\times W \right)=2\times\Delta A_{T}\div\Delta A_{S}$$

where C is concentration of H2O2 standard solution, 2 μmol/mL, Vs is sample volume, 0.25 ml, W is sample weight, g, Veis extraction volume, 1 ml.

1.2. Procedure S2: ATP content

Sample preparation: Collecting 0.1 g sample. The volume of Extract solution (mL) is 500~1000:1. It is suggested to add 1 mL of Extract reagent to 5 million bacteria or cells. Use ultrasonication to split bacteria and cell (placed on ice, ultrasonic power 200 W, working time 2 s, interval 1 s, repeat for 20 times). Centrifuge at 10000×g for 10 minutes at 4℃ to remove insoluble materials and take the supernatant into another EP tube. Add 500 μL of chloroform into the supernatant and shock blending. Centrifuge at 10000×g for 3 minutes at 4℃ to remove insoluble materials and take the supernatant on ice before testing.

Detect procedure:

1. Preheat spectrophotometer or microplate reader for 30 minutes, adjust the wavelength to 340 nm, and set it to zero with distilled water.

2. Dilute the 10 μmol/mL standard solution 16 times to 0.625 μmol/mL standard with distilled water.

3. Add reagents with the following list:

**Supplementary method Table 2.**

| Reagent (μL) | Test Tube (T) | Standard Tube (S) |
| --- | --- | --- |
| sample | 20 | 0 |
| Standard solution | 0 | 20 |
| Reagent Ⅰ | 128 | 128 |
| working solution | 52 | 52 |

Mix and timing, detect the absorbance at 340 nm at the tie of 10 s and record as A1 (10 S). Then place the cuvette with the reaction solution in a 25℃ water bath or incubate for 3 min. Take it out and wipe it clean, then immediately measure the absorbance at 190 s of final reaction which is recorded as A2. ΔA_T_=A2_T_-A1_T_, ΔA_S_=A2_S_-A1_S_

Calculation:

ATP (μmol/g fresh weight)=∆A_T÷(∆A_S÷C_S )×V_e÷W=0.625×∆A_T÷∆A_S÷W

where Cs is standard concentration, 0.625 μmol/mL, Ve is extracting volume, 1 mL, W is sample weight, g.

1.3. Procedure S3: Protein content

Completely dissolve BSA standard, dilute 10 μL to 250 μL, the final concentration of 0.2mg/ml. Dilution buffer was depended on the measured protein sample. For the sake of simplicity, suggested to use 0.9% NaCl or PBS. 5×G250 mix well before use, then 1ml 5×G250 diluted with 4ml ddH_2_O. 1×G250 solution can save one week at 4℃. The standard according to 0, 2, 4, 6, 8, 12, 16, and 20 μL respectively added to 96well plates, add PBS dilution to complement 20 μL.

Dilute the sample (prepare a few gradients, such as 2 times, 4 times, 8 times dilution), and add 20 μL sample to 96 well plates. To avoid errors, sample points need set after the standard line of 1/2. Add 200 μL diluted 1×G250 to each well, room temperature for 3-5 minutes. Determination of absorbance. According to the standard curve calculate the sample protein concentration.

1.4. Procedure S4: Total reducing sugar content

Extraction of reducing sugar: Collect the bacteria or cells into the centrifuge tube, and discard the supernatant after centrifugation. For bacteria or cells (0.1 g), the volume (mL) of Reagent Ⅰ is 500~1000:1 (It is suggested to add 2 mL of Reagent Ⅰ to 10 million of bacteria or cells), ultrasonic broke bacteria or cells (ice bath, power of 20% or 200 W, ultrasound for 3 s, interval of 10 s, repeat 30 times). Transfer to the covered centrifuge tube (to prevent water loss during heating), water bath at 80℃ for 40 minutes, and during which shake for 8-10 times. Centrifuge at 8000×g for 10 minutes at 25℃, and take the supernatant for determination.

Determination procedure:

1. Preheat the spectrophotometer/microplate reader for 30 minutes, adjust the wavelength to 540 nm, and adjust zero with distilled water.

2. Standard preparation: Dilute the standard with distilled water to 0.6、0.5、0.4、0.3、0.2、0.1 mg/mL.

3. Add the following reagents successively into the EP tube:

**Supplementary method Table 3.**

| Reagent (μL) | Contrast Tube (C) | Test Tube (T) | Standard Tube (S) | Blank Tube (B) |
| --- | --- | --- | --- | --- |
| sample | 175 | 175 | 0 | 0 |
| Standard working solution | 0 | 0 | 175 | 0 |
| Reagent Ⅱ | 0 | 125 | 125 | 125 |
| Distilled water | 125 | 0 | 0 | 175 |

Mix, heat in boiling water bath for 5 minutes (cover tightly to prevent water loss), cool to room temperature immediately after taking out, and mix well. Take 200 μL of the reaction solution to a micro glass cuvette or 96 well plate, and read the absorbance values of standard tube, contrast tube, test tube, and blank tube at 540 nm. Calculate ΔA=A_T_-A_C_.

Calculation of reducing sugar content:

1. Standard curve: According to the concentration and absorbance of the standard tube (AS-AB), establish the standard curve, x is the absorbance value, y is the concentration of the standard (mg/mL). Calculate the content of reducing sugar in the sample according to the standard curve. Take ΔA (A_T_-A_C_) into x to obtain y value by calculating.

2. Calculate by Sample fresh weight:

$$Reducing sugar content \left( \mu mol/g fresh weight \right)=1000\times y\times V1\div W=1000\times y\div W$$

where 1000 is the unit conversion coefficient, 1 mg/mL=1000 μg/mL, V1 is Add the volume of Reagent I, 1 mL, W is sample fresh weight, g.

1.5. Procedure S5: Electron transport chain complex Ⅰ activity

Complex extraction:

1. Collecting 0.1 g of tissue or 5 million cells, add 1 mL of extract solution, grinding on ice with mortar/homogenizer.

2. Centrifuge at 600×g for 10 minutes at 4℃. Take the supernatant to another tube and centrifuge at 11000 g for 15 minutes at 4℃.

3. Supernatant is cytoplasmic extract. It can be used to detect complex Ⅰ that leaks from mitochondria, which shows the effect of mitochondrial extraction.

4. Add 400 μL of extract solution to the sediment, splitting with ultrasonic (power 20%, work time 5 s, interval 10 s, repeat 15 times), used to detect the enzyme activity of complex Ⅰ and protein content.

Determination:

1. Preheat ultraviolet spectrophotometer for 30 minutes, adjust the wavelength to 340 nm, set zero with distilled water.

2. Preheat Reagent I at 37℃ (mammal) or 25℃ (other species) for 15 minutes.

3. Add the following reagents in 1 mL quartz cuvette:

**Supplementary method Table 4.**

| Reagent | Test tube (T) |
| --- | --- |
| Sample (μL) | 50 |
| Reagent I (μL) | 770 |
| Working solution (μL) | 100 |
| Reagent IV (μL) | 80 |

Mix thoroughly and timing, detect the absorbance at 340 nm at the time of 10 seconds record as A1 (10 s). then try to react accurately in 37℃ (mammal) or 25℃ (other species) environment for 2 minutes. Take it out and wipe it clean, immediately measure the absorbance of final reaction which recorded as A2 (130 s). ΔA=A1-A2.

Calculation: One unit of enzyme activity is defined as the amount of enzyme catalyzes the consumption of 1 nmol NADH per minute for every milligram of tissue protein.

$$\mathrm{Complex} Ⅰ\mathrm{activity} \left( U/mg prot \right)=\left[ \Delta A\times V_{rv}\div\left( \varepsilon\times d \right)\times{10}^{9} \right]\div\left( V_{S}\times C_{pr} \right)\div T=1608\times\Delta A\div C_{pr}$$

where ε is NADH molar extinction coefficient, 6.22×10^3^ L/mol/cm, d is light path of cuvette, 1 cm, Vrv is total reaction volume,10-3 L, Vs is sample volume (mL), 0.05 mL, Cpr is sample protein concentration (mg/mL), T is reaction time (min), 2 minutes.

1.6. Procedure S6: Electron transport chain complex Ⅱ activity

Complex II extraction:

1. Collecting 0.1g of tissue or 5 million cells, add 1ml of extract solution and grind on ice with mortar/homogenizer.

2. Centrifuge at 600 g and 4℃ for 10min. Discard the precipitate and transfer supernatant to another tube, centrifuge at 11000 g and 4℃ for 15 min.

3. The supernatant, i.e. cytoplasmic extract, can be used to determine the complex Ⅱ leaking from mitochondria, this step can show the effect of mitochondrial extraction.

4. Add 400 μL extraction solution to sediment, splitting with ultrasonication (power 20%, work time 5s, interval 10s, repeat 15 times), used to detect complex Ⅱ activity and protein content.

Determining step:

1. Preheat spectrophotometer/microplate for 30 min, adjust the wavelength to 605 nm, and set the counter to zero with distilled water.

2. Making working solution: mix reagent 2 and reagent 3 as ratio of 1:1 before use. Prepared when the solution will be used.

3. Preheat reagent1 at 37℃ (mammal cell), 25℃ (other species) for 15 min.

4. Add the following reagents in micro glass cuvette:

**Supplementary method Table 5.**

| Reagent name (μL) | Test tube A1 |
| --- | --- |
| Sample | 10 |
| Reagent 1 | 150 |
| Working solution | 20 |
| Reagent 4 | 20 |

Add the above reagent to the micro glass cuvette, mix thoroughly, and detect absorbance at 10 s (A1). Put cuvette and react solution together in 37℃ (mammal) or 25℃ (other species) water bath for 2 min, then take cuvette quickly, dry, and detect absorbance at 2 min (A2), ΔA=A1-A2.

Calculation: One unit of enzyme activity is defined as the amount of enzyme that catalyzes the consumption of 1nmol of 2, 6-dichlorindolepheno per mg of tissue protein every minute.

$$\mathrm{Complex} Ⅱ\mathrm{activity} \left( nmol/min/mg prot \right)=\left[ \Delta A\times V_{rv}\div\left( \varepsilon\times d \right)\times{10}^{9} \right]\div\left( V_{S}\times C_{pr} \right)\div T=476.2\times\Delta A\div C_{pr}$$

where ε is 2, 6-dichlorindolepheno molar extinction coefficient, 2.1×104 L/mol/cm, d is light path of cuvette, 1 cm, Vrv is total reaction volume, 2×10-4 L, Vs is sample volume (mL), 0.01 mL, Cpr is sample protein concentration (mg/mL), T is reaction time (min), 2 min.

1.7. Procedure S7: Electron transport chain complex Ⅲ activity

Complex III extraction:

1. Collecting 0.1g of tissue or 5 million cells, add 1 ml extract solution and grind on ice with mortar/homogenizer.

2. centrifuge at 600 g and 4℃ for 10 min. Discard the precipitate and transfer supernatant to another tube, centrifuge at 11000 g and 4℃ for 15 min.

3. The supernatant, i.e. cytoplasmic extract, can be used to determine the complex III leaking from mitochondria, this step can show the effect of mitochondrial extraction.

4. Add 200 μL of extraction solution to sediment, splitting with ultrasonication (power 20%, work time 5 s, interval 10 s, repeat 15 times), used to detect complex III activity and protein content.

Determining step:

1. Preheat spectrophotometer/microplate reader for 30 min, adjust the wavelength to 550 nm, and set the counter to zero with distilled water.

2. Making working solution: Transfer one bottle of reagent2 to one bottle of reagent1 to dissolve thoroughly before use, and unused reagent at 4℃ for one week.

3. Add the following reagents in 1 ml cuvette:

**Supplementary method Table 6.**

| Reagent name (μL) | Test tube (At) | Control tube (Ac) |
| --- | --- | --- |
| Working solution | 160 | 160 |
| Reagent 3 | 20 | - |
| Accurately incubate for 2 min at 37 °C (mammal) or 25 °C (other species), then add separately as follows | | |
| Sample | 20 | 20 |
| Distilled water |  | 20 |

Add the above reagent to the cuvette, mix thoroughly, and detect absorbance at 10 s (At1 and Ac1). Put cuvette and react solution together in 37℃ (mammal) or 25℃ (other species) water bath for 2 min, then take cuvette quickly, dry, and detect absorbance at 2 min (At2 and Ac2), ΔA=(At2-At1)-(Ac2-Ac1)

Calculation: One unit of enzyme activity is defined as the amount of enzyme that catalyzes the consumption of 1 nmol of reduced cytochrome C per mg of tissue protein every minute.

$$\mathrm{Complex} Ⅲ\mathrm{activity} \left( U/mg prot \right)=\left[ \Delta A\times V_{rv}\div\left( \varepsilon\times d \right)\times{10}^{9} \right]\div\left( V_{S}\times C_{pr} \right)\div T=261\times\Delta A\div C_{pr}$$

where ε is reduced cytochrome C molar extinction coefficient, 19100 L/mol/cm, d is light path of cuvette, 1 cm, Vrv is total reaction volume, 0.0002 L, Vs is sample volume (mL), 0.02 mL, Cpr is sample protein concentration (mg/mL), T is reaction time (min), 2 min.

1.8. Procedure S8: Mitochondrial respiratory chain complex Ⅳ activity

Complex extraction:

1. Collecting 0.1 g of tissue or 5 million cells, add 1 mL of extract solution, grinding on ice with mortar/homogenizer. Centrifuge at 600×g for 10 minutes at 4℃.

2. Take the supernatant to another tube and centrifuge at 11000×g for 15 minutes at 4℃.

3. The supernatant can used to detect complex Ⅳ that leaking from mitochondria, which shows the effect of mitochondrial extraction.

4. Add 400 μL of extract solution to the sediment, splitting with ultrasonic (power 20%, work time 5 s, interval 10 s, repeat 15 times), used to detect the enzyme activity of complex Ⅳ and protein content.

Determination procedure:

1. Preheat microplate reader or spectrophotometer for 30 minutes, adjust the wavelength to 550 nm, and set zero with distilled water.

2. Preheat working solution at 37℃ (mammal) or 25℃ (other species) for 15 minutes. Unused reagents can be stored for one week at 4℃.

3. Add the following reagents in 1 mL glass cuvette:

**Supplementary method Table 7.**

| Reagent | Test tube (T) | Blank tube (B) |
| --- | --- | --- |
| Sample (μL) | 10 | - |
| Distilled water | - | 10 |
| Working solution (μL) | 200 | 200 |

Mix thoroughly and timing, detect the absorbance of initial and final reaction at 550 nm, and record as A1 (0 s) and A2 (1 min) respectively. ΔA(T)=A2(T)-A1(T), ΔA(B)=A2(B)-A1(B).

Calculation: one unit of enzyme activity is defined as the amount of enzyme catalyzes the degradation of 1nmol of reduced cytochrome C per minute for every milligram tissue protein.

$$\mathrm{Complex} Ⅳ\mathrm{activity} \left( U/mg prot \right)=\left[ \Delta A\times V_{rv}\div\left( \varepsilon\times d \right)\times{10}^{9} \right]\div\left( V_{S}\times C_{pr} \right)\div T=1099\times\Delta A\div C_{pr}$$

where ε is cytochrome C molar extinction coefficient, 1.91×10^4^ L/mol/cm, d is light path of cuvette, 1 cm, Vrv is total reaction volume,2.1×10^-4^ L, Vs is sample volume (mL), 0.01 mL, Cpr is sample protein concentration (mg/mL), the protein concentrate of the sample needs to be determined by yourself and our PC0020 BCA Protein Assay Kit is recommended, T is reaction time (min), 1 minute.

1.9. Procedure S9: Catalase (CAT) activity

Sample preparation: Collect bacteria or cells into the centrifuge tube, after centrifugation discard supernatant. It is suggested to add 1 mL of extraction reagent to 5 million bacteria or cells. Use ultrasonication to split bacteria and cells (place on ice, ultrasonic power 200 W, working time 3 seconds, interval 10 seconds, repeat 30 times). Centrifuge at 8000×g for 10 minutes at 4℃ to remove insoluble materials and take the supernatant on ice for testing.

Determination procedure:

1. Preheat the spectrophotometer for more than 30 minutes, adjust the wavelength to 240 nm, and set zero with distilled water.

2. Preheat CAT working reagent in water bath at 37℃ (mammals) or 25℃ (other species) for 10 minutes.

3. Add 190 µL of CAT working reagent and 10 µL of sample in micro quartz cuvette. Immediately mix and detect the absorbance at 240 nm at the initial time (A1) and the absorbance after reaction for 1 minute (A2), calculate ΔA=A1-A2.

Calculation: One unit of enzyme activity is defined as the amount of enzyme catalyzes the degradation of 1μmol of CAT in the reaction system per minute for every milligram protein.

$$\mathrm{CAT} \left( U/mg prot \right)=\left[ \Delta A\times V_{rv}\div\left( \varepsilon\times d \right)\times{10}^{9} \right]\div\left( V_{S}\times C_{pr} \right)\div T=764.5\times\Delta A\div C_{pr}$$

where Vrv is reaction total volume, 2×10^-4^ L, ε is molar extinction coefficient, 43.6 L/mol/cm, d is light path of cuvette, 1 cm, Vs is sample volume,0.01 mL, T is reaction time, 1 minute, Cpr is sample protein concentration, mg/mL, 10^6^ is unit conversion factor, 1 mol=10^6^ μmol.

1.10. Procedure S10: Peroxidase (POD) activity

Sample preparation: Collecting bacteria or cells into the centrifuge tube, the supernatant is discarded after centrifugation. It is suggested to take about 5 million bacteria/cell and add 1 mL of extract solution. Bacteria and cell are broken by ultrasonication (Power: 20%, work time 3 s, interval 10 s, repeat 30 times). Centrifuge at 8000 rpm for 10 minutes at 4℃, the supernatant is used for test.

Determination procedure:

1. Preheat Spectrophotometer/microplate reader for 30 minutes, adjust wavelength to 470 nm, and set zero with distilled water.

2. Reagent I, Reagent II and Reagent III are placed at 37℃ (mammal) or 25℃ (other species) for 10 minutes before determination.

3. Add reagents with the following list:

**Supplementary method Table 8.**

| Name of reagent (µL) | Test tube |
| --- | --- |
| Reagent I | 120 |
| Reagent II | 30 |
| Reagent III | 30 |
| Distilled water | 60 |
| Sample | 5 |

The above reagents are added into EP tubes in sequence, immediately mixed, and timed. Then 200 μL of the mixed solution is immediately transferred to a micro glass cuvette. The absorbance values A1 for 30 s and A2 for 90 s at 470 nm are recorded, ΔA＝A2-A1.

Calculations: One unit of enzyme activity is defined as the amount of enzyme catalyzes the absorbance of 0.01 change at 470 nm in the reaction system per minute for every milligram protein.

$$\mathrm{POD} \left( U/mg prot \right)=\Delta A\times V_{rv}\div\left( V_{SV}\times C_{pr} \right)\div0.01\div T=4900\times\Delta A\div C_{pr}$$

where Vrv is total reaction volume,0.245 mL, Vsv is total supernatant volume, 0.005 mL, T is reaction time, 1 minute, Cpr is sample protein concentration, mg/mL.


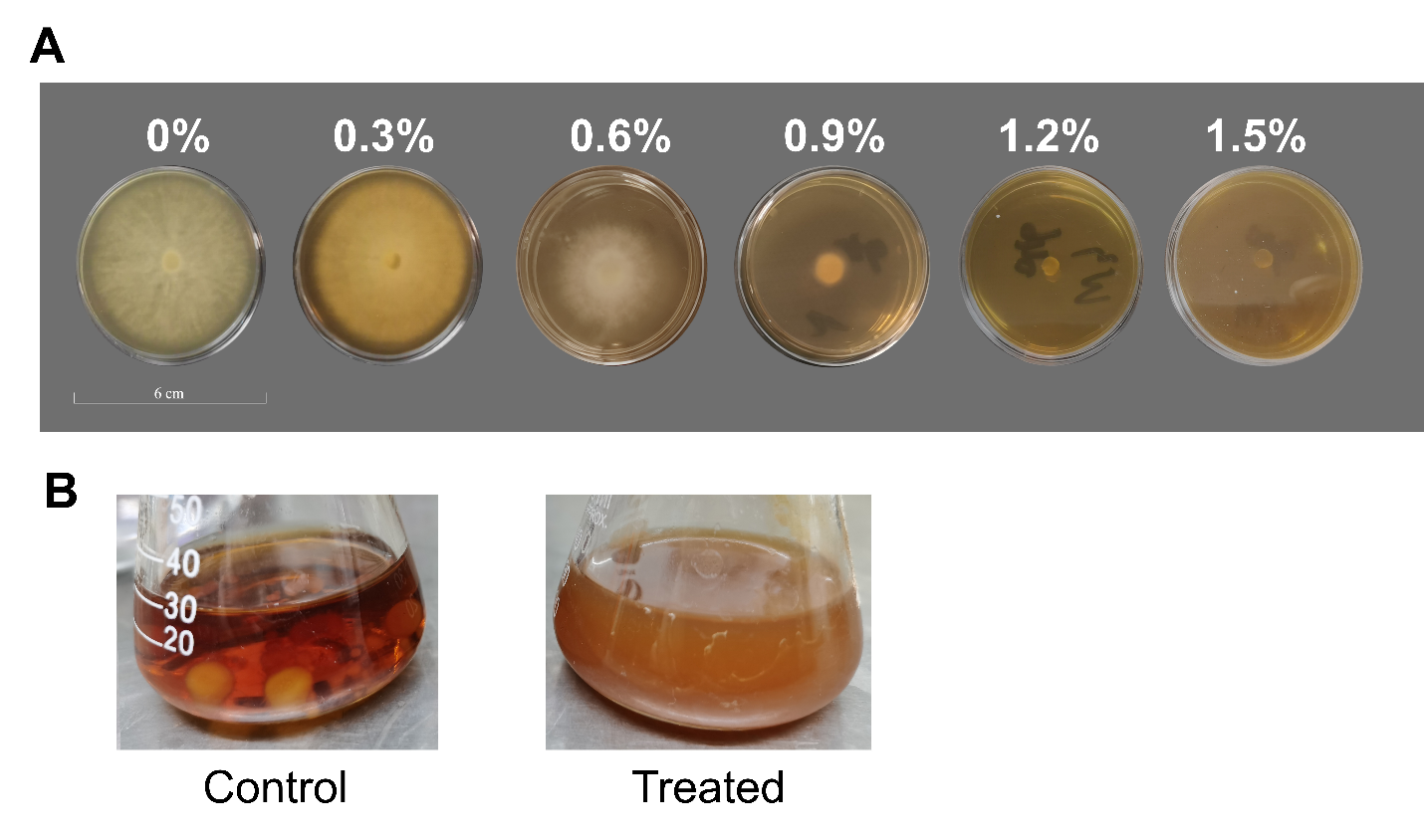


Figure S1. The inhibition effect of HTL-AP on *R*. *solani*. a, Gradient plate antifungal experiment of HTL-AP. b, Effect of HTL-AP on liquid culture of fungi.


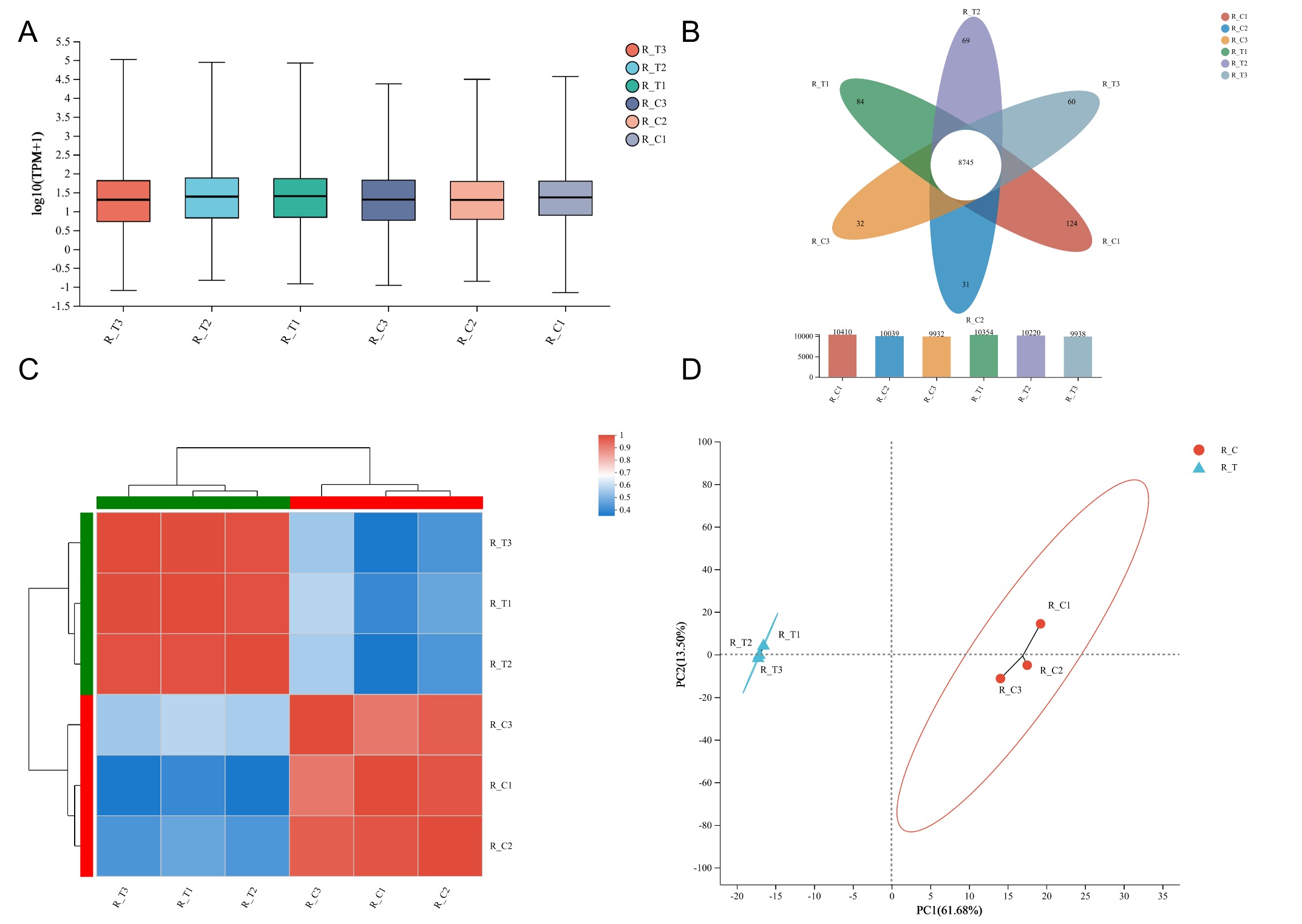


Figure S2. Gene expression analysis of *R*. *solani* treated by HTL-AP.

RSEM software was used to standardize the gene expression levels of the test group and the control group by TPM method. According to the results of the box graph (A), the overall expression level of unigene in the fungal transcriptome of the treatment group was low. A total of 11490 expressed unigenes were detected, and according to the Venn plot (B), 8121 unigenes were expressed in all samples. The correlation analysis results show that the correlation coefficients between samples within the group are all above 0.92 (C), indicating that each biological repeated sample has a relatively similar expression pattern, ensuring that the sequencing and sampling results of this time can be used for subsequent analysis. PCA analysis was conducted on the expression levels of all genes, and the results showed that the microalgae HTL-AP treatment group and the control group were clearly distinguished. Three biological duplicate samples within each group were clustered together (D), indicating significant differences between groups and high similarity within the group. Next, DESeq2 software was used to compare the differentially expressed genes between the experimental group and the control group (Padjust<0.05 and | log2FC | ≥ 2).


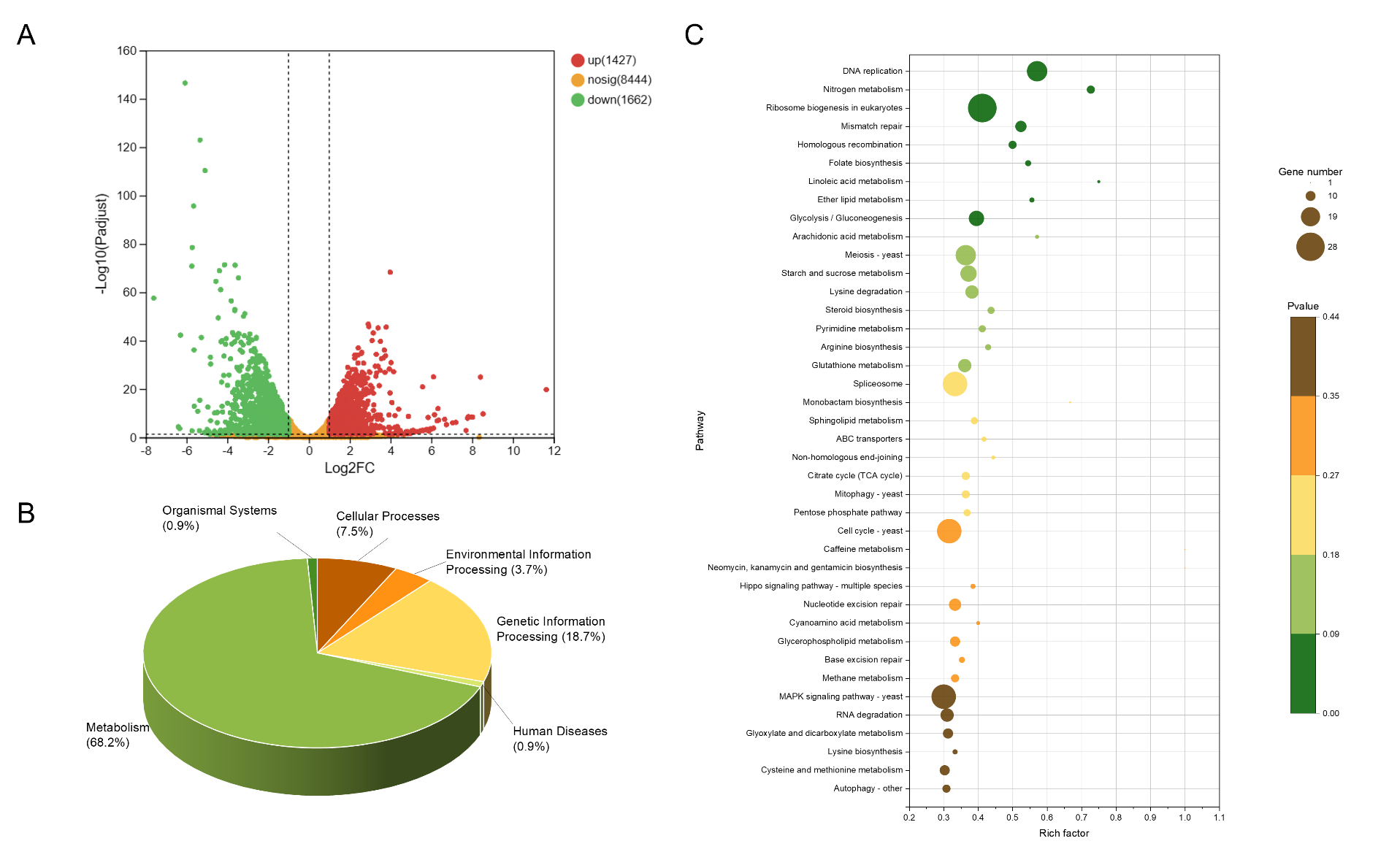


Figure S3. DEGs analysis of *R*. *solani* treated by HTL-AP.

A total of 3089 different expressed genes (DEGs) were identified after HTL-AP treatment, among which 1662 DEGs were down-regulated and 1427 DEGs were up-regulated (A). Then these DEGs were classified into six categories using KEGG enrichment analysis (B). The top 40 most enriched pathways indicated that HTL-AP mainly acted on metabolism, genetic information processing and cellular processes. Among enriched DEGs, 42 DEGs were related to cell growth and death, 56 DEGs were involved in carbohydrate metabolism, 65 DEGs were linked to protein synthesis and 16 DEGs pertained to energy metabolism (C), which were consistent with the experimental results in the previous section. The detailed DEGs analysis results are shown in **Data S5-S7**.


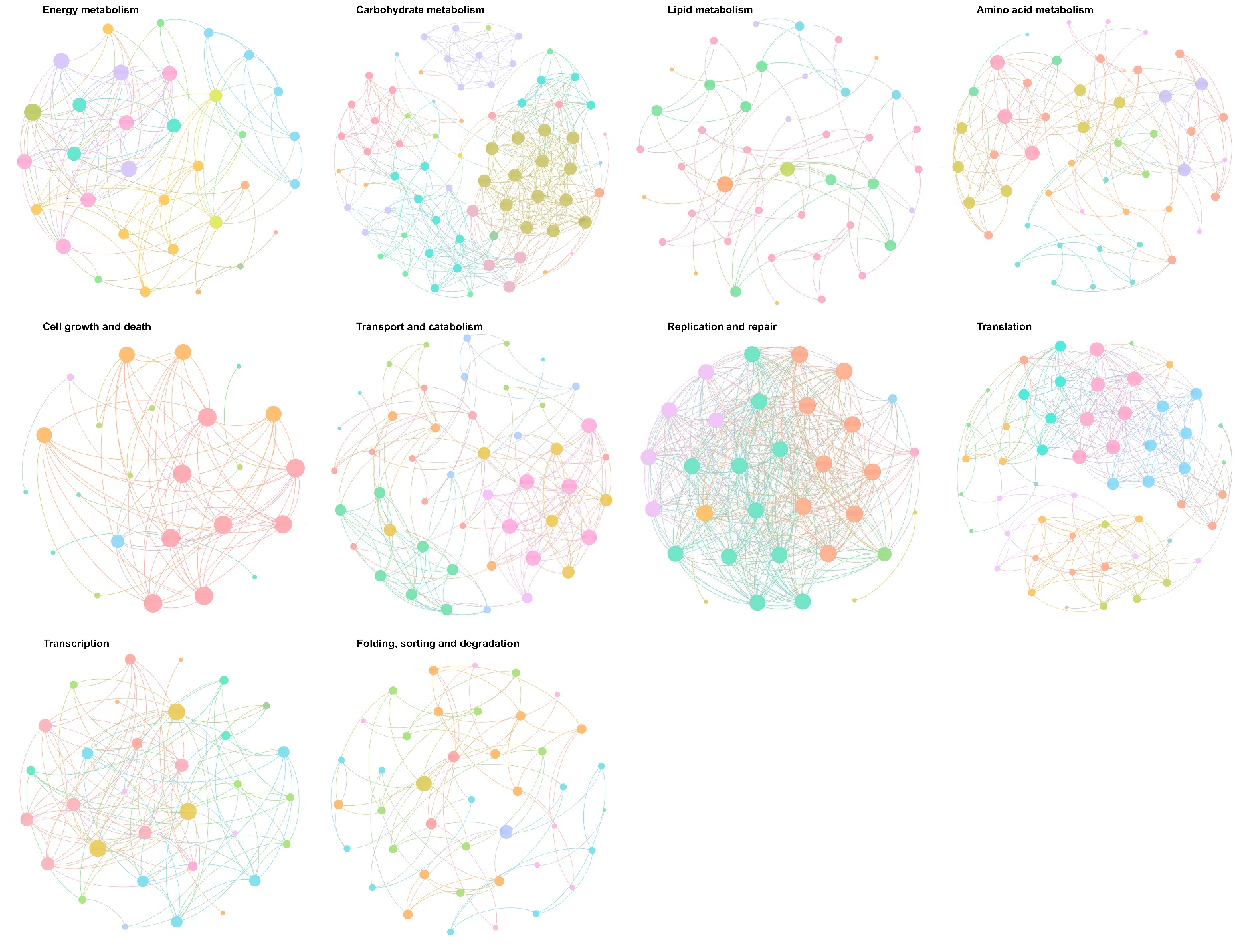


Figure S4. Co-expression network construction of EDGs.

We further use DEGs of metabolism, genetic information processing and cellular processes categories to construct co-expression network. All second categories cell in cellular processes and genetic information processing (cell growth and death, transport, and catabolism, replication and repair, translation, transcription, and folding, sorting, and degradation) were used to establish gene expression network, respectively. And in metabolism, some second categories contained fewer genes, so only four second pathways (energy metabolism, carbohydrate metabolism, lipid metabolism and amino acid metabolism) with more genes were chose to establish gene expression network. Subsequently, the genes with top 20% degree were selected as hub gene ^[1]^. The detailed gene information is situated in **Table S4**.


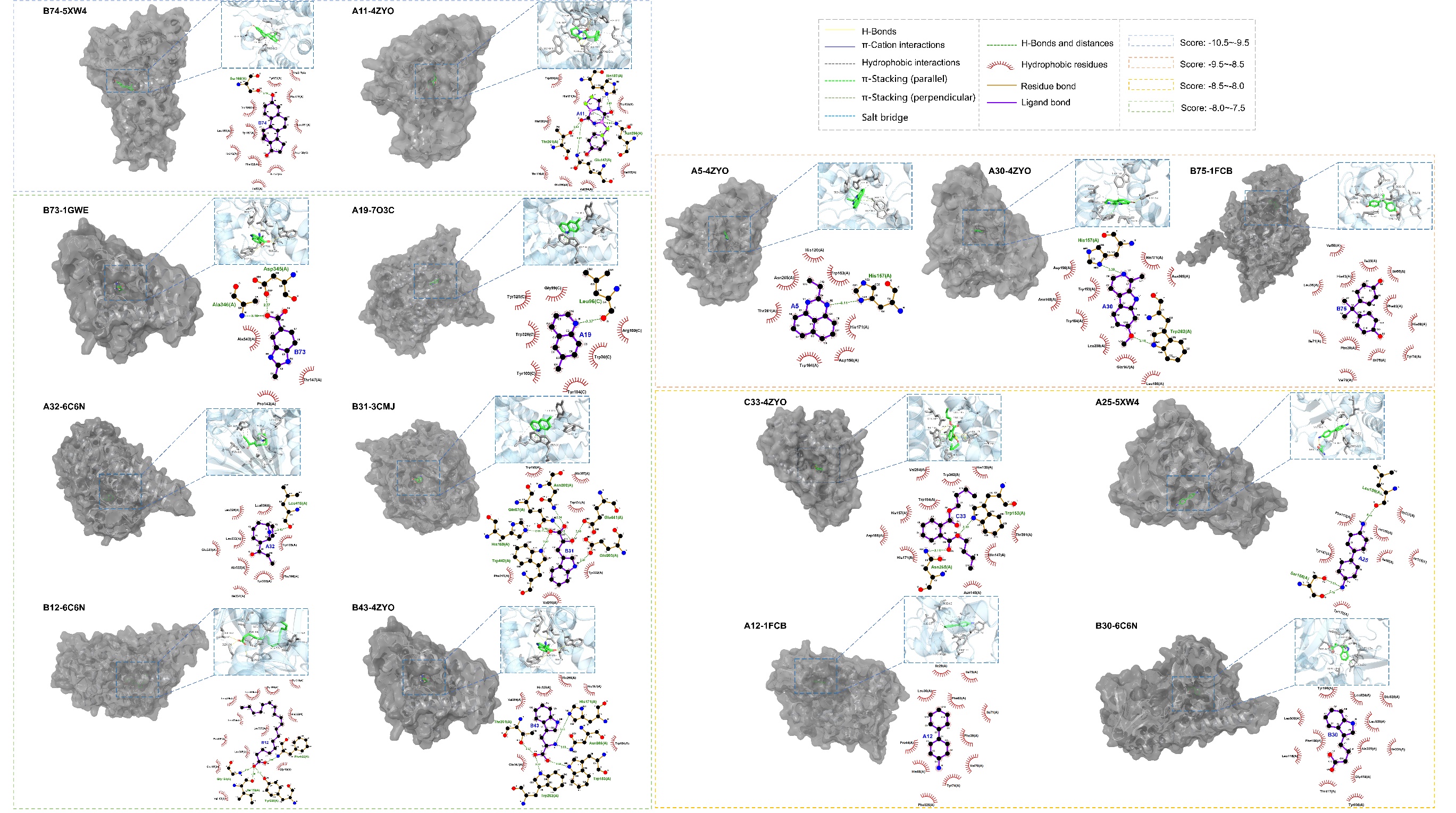


Figure S5. Visualization of 15 extracted substance-protein complexes.

We extracted 15 complexes from different binding affinity segments and analyzed the interactions between substances and target proteins, B74-5XW4 (Estrone - Tyrosine-protein phosphatase, -10.5 kcal/mol), A11-4ZYO (3-[(4-hydroxyphenyl)methyl]-octahydropyrrolo[1,2-a]pyrazine-1,4-dione - Stearoyl-CoA desaturase, -9.8 kcal/mol), A5-4ZYO (2-ethyl-1H-perimidine - Stearoyl-CoA desaturase, -9.1 kcal/mol), A30-4ZYO (Harmaline - Stearoyl-CoA desaturase, -8.8 kcal/mol), B75-1FCB (Bisphenol A - L-lactate dehydrogenase, -8.8 kcal/mol), C33-4ZYO (Dibutyl phthalate - Stearoyl-CoA desaturase, -8.3 kcal/mol), A25-5XW4 (Benzidine - Tyrosine-protein phosphatase, -8.0 kcal/mol), A12-1FCB (4-Aminobiphenyl - L-lactate dehydrogenase, -8.2 kcal/mol), B30-6C6N (Indole-3-butyric acid - Squalene monooxygenase, -8.1 kcal/mol), B73-1GWE (2-methyl-1H-benzimidazole-5-carboxylic acid - Catalase, -7.7 kcal/mol), A19-7O3C (6-Methylquinoline - Mitochondrial respiratory chain complex Ⅲ, -7.7 kcal/mol), A32-6C6N (Homo-anatoxin - Squalene monooxygenase, -7.5 kcal/mol), B31-3CMJ (Indole-3-lactic acid - Beta-glucosidase,-7.8 kcal/mol), B12-6C6N (9(Z),11(E)-Conjugated linoleic acid - Squalene monooxygenase, -7.9 kcal/mol) and B43-4ZYO (DL-Tryptophan - Stearoyl-CoA desaturase, -7.9 kcal/mol).

Protein 5XW4 is one of the key enzymes involved in the regulation of mitotic exit, which can let the cell return to G1 phase.^[2]^ The hydroxyl on B74 benzene ring and residue Ser166 formed a hydrogen bond. A25 could also occupy the active site of 5XW4, its two nitrogen atoms formed three hydrogen bonds with residues Leu156 and Ser166 respectively. It confirmed components would insert in the non-polar pocket of 5XW4 to competitively inhibit the phosphopeptide substrate,^[3]^ which would block mitotic cycle and inner mitotic process to stay in the G2/M phase.^[4]^ Protein 4ZYO can maintain the balance between saturated and unsaturated lipids in cells.^[5]^ The carbonyl on nitrogen heterocycle of A11 formed three hydrogen bonds with residues His157, Thr261 and Gln147, and the nitrogen heterocycle also formed two hydrogen bonds with residues Asn265. Compound A5, A30, C33 and B43 also inserted into the hydrophobic channel of 4ZYO. And the benzene ring of A5 formed three parallel-displaced π-π stacking interactions with residue Trp153, and also formed two T-shaped π-π stacking interactions with residue Sih171, its nitrogen heterocycle formed one hydrogen bond with residues His157. The oxygen and nitrogen of A30 respectively formed hydrogen bonds with residue Trp262 and His157, and tis nitrogen-containing heterocycles formed a parallel-displaced π-π stacking interaction with residue Trp153. The oxygen of C33 formed two hydrogen bonds with residue Trp153 and Asn265, its benzene ring formed two parallel-displaced π-π stacking interactions with residue Trp153 and a perpendicular-displaced π-π stacking interaction with residue His171, and also formed a salt bridge with residue His171. The two oxygens of B43 formed three hydrogen bonds with residue Thr261, Trp262 and Trp153, its nitrogen formed two hydrogen bonds with residue His171 and Asn265, and its benzene ring formed a parallel-displaced and a perpendicular-displaced π-π stacking interaction with residues Trp153 and Trp184. Hence, substances would compete with stearyl-CoA to insert into the hydrophobic channel of 4ZYO, resulting in disorder of lipid metabolism pathway.^[6]^

Protein 1FCB is part of a secondary electron transport chain, composition A12 and B75 inserted into the heme group catalytic domain in subunit 1 of 1FCB blocking the reduction of cytochrome c.^[7]^ Protein 6C6N is flavin adenosine dinucleotide (FAD)–dependent epoxidase, which can catalyze squalene to 2,3(S)-oxidosqualene a committed step in cholesterol biosynthesis.^[8]^ Substances B30, B12, and A32 occupied the open end of the pocket proximal to FAD in 6C6N. The benzene ring of B30 formed a parallel-displaced π-π stacking interaction with residue Tyr195, the two oxygens of B12 formed four hydrogen bonds with residue Phe166, Gly164, Ile162 and Tyr335, and the nitrogen of A32 formed a hydrogen bond with residue Leu416. In hydrophobic channel of 1GWE, the carbonyl of B73 formed two hydrogen bonds with residues Ala346 and Asp346 respectively. The benzene ring of A19 formed two parallel-displaced π-π stacking interactions with residue Trp30, and its nitrogen heterocycle also formed a parallel-displaced π-π stacking interaction with residue Trp30. Protein 1ZLT belongs to serine/threonine protein kinases which can affect cell proliferation, differentiation and other functions.^[9]^ The G-loop of 1ZLT played a major role in its catalytic regulation and A50 could insert it, and its oxygen formed a hydrogen bond with residues Ser147, inhibiting catalytic activity and obstructing DNA damage repair.^[10]^ ^[11]^ Protein 3CMJ belongs to β-Glucosidase, used to hydrolyze β-1,4-glucoside bond.^[12][13]^ B31 formed hydrophobic interactions in the hydrophobic channel of 3CMJ. Its three oxygens formed 6 hydrogen bonds with residues His158, Trp442 Gln57, Glu441 and Asn202, inhibiting the carbohydrate metabolism which was consistent with the results of fungus inhibition test.


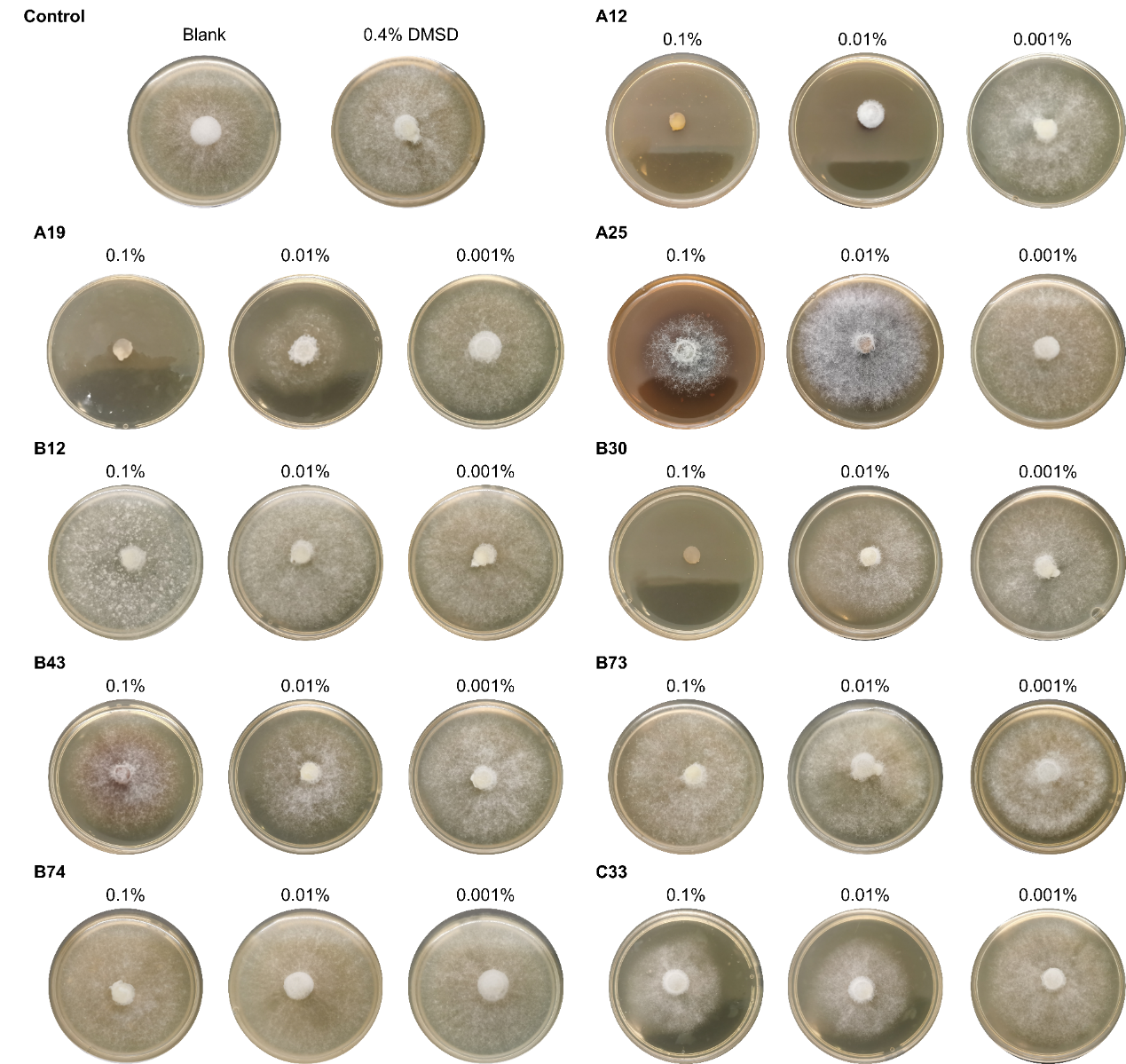


Figure S6. Inhibition capacity of potential antifungal components with different concentrations.


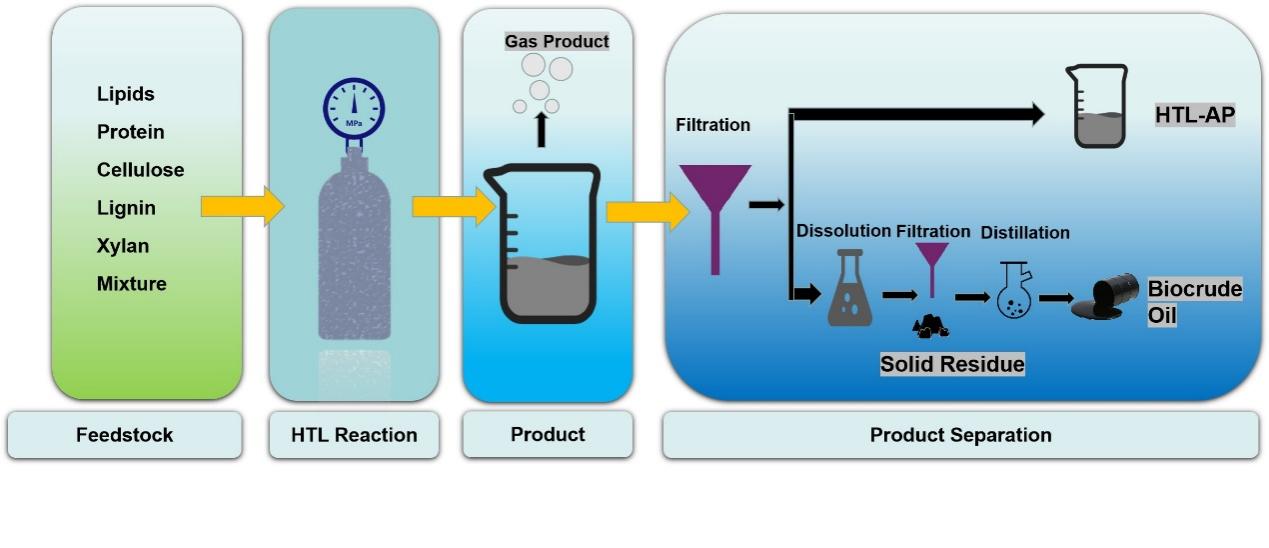


Figure S7. Procedure of HTL-AP preparation.

**Table S1.** Biochemical analysis of *Spirulina* sp. and HTL-AP.

| Parameter | Raw material | Parameter | HTL-AP |
| --- | --- | --- | --- |
| Ash (d.w., %)a) | 6.9±0.5 | pH | 8.66±0.01 |
| Lipid (d.w., %) | 6.9±0.5 | Conductivity (mS/cm) | 41.6±0.12 |
| Protein (d.w., %) | 63.1±0.9 | NH4-N (g/L) | 4.33±0.34 |
| Carbohydrateb (d.w., %)b) | 23.8 | TN (g/L) | 6.73±0.42 |
| C (d.w., %) | 42.0±1.9 | COD (g/L) | 48.46±7.34 |
| H (d.w., %) | 7.2±0.2 | Al (mg/kg) | NDc) |
| N (d.w., %) | 10.3±0.7 | Ca (mg/kg) | 67.3 |
| Al (mg/kg) | 99.1 | Cu (mg/kg) | ND |
| Ca (mg/kg) | 2540.2 | Fe (mg/kg) | ND |
| Cu (mg/kg) | 3 | K (mg/kg) | 987.9 |
| Fe (mg/kg) | 575.3 | Mg (mg/kg) | 23.2 |
| K (mg/kg) | 7701.7 | Na (mg/kg) | 203.5 |
| Mg (mg/kg) | 2906.1 | Zn (mg/kg) | ND |
| Na (mg/kg) | 1544.9 |  |  |
| Zn (mg/kg) | 8.4 |  |  |

^a)^ (Dry weight basis); ^b)^ (Carbohydrate (d.w.%) =100-(Ash+Lipid+ Protein) (d.w.%)); ^c)^ (Not detected).

Table S2. Degree of DEGs in co-expression network.

| KEGG first categories | KEGG second categories | Genes | Degree | Hub genes |
| --- | --- | --- | --- | --- |
| Metabolism | Energy metabolism | TRINITY_DN6823_c0_g1 | 13 | √ |
|  |  | TRINITY_DN4502_c0_g2 | 12 | √ |
|  |  | TRINITY_DN6076_c0_g1 | 12 |  |
|  |  | TRINITY_DN6766_c0_g1 | 12 |  |
|  |  | TRINITY_DN6570_c0_g1 | 11 | √ |
|  |  | TRINITY_DN3549_c0_g5 | 11 |  |
|  |  | TRINITY_DN5144_c0_g1 | 11 | √ |
|  |  | TRINITY_DN5144_c0_g2 | 11 | √ |
|  |  | TRINITY_DN6120_c0_g1 | 11 | √ |
|  |  | TRINITY_DN5009_c0_g2 | 10 |  |
|  |  | TRINITY_DN6448_c0_g1 | 10 |  |
|  |  | TRINITY_DN4502_c0_g1 | 10 |  |
|  |  | TRINITY_DN2340_c0_g2 | 9 |  |
|  |  | TRINITY_DN5840_c0_g1 | 9 |  |
|  |  | TRINITY_DN1337_c0_g1 | 7 |  |
|  |  | TRINITY_DN1445_c0_g1 | 7 |  |
|  |  | TRINITY_DN2178_c0_g1 | 7 |  |
|  |  | TRINITY_DN244_c0_g1 | 7 |  |
|  |  | TRINITY_DN432_c0_g5 | 7 |  |
|  |  | TRINITY_DN4544_c0_g1 | 7 |  |
|  |  | TRINITY_DN2380_c0_g1 | 7 |  |
|  |  | TRINITY_DN1234_c0_g1 | 6 |  |
|  |  | TRINITY_DN1500_c0_g3 | 6 |  |
|  |  | TRINITY_DN2592_c0_g1 | 6 |  |
|  |  | TRINITY_DN5859_c0_g1 | 6 |  |
|  |  | TRINITY_DN6073_c0_g1 | 6 |  |
|  |  | TRINITY_DN282_c0_g1 | 5 |  |
|  |  | TRINITY_DN1601_c0_g1 | 4 |  |
|  |  | TRINITY_DN2483_c0_g5 | 4 |  |
|  |  | TRINITY_DN3776_c0_g3 | 4 |  |
|  |  | TRINITY_DN6536_c0_g1 | 3 |  |
|  |  | TRINITY_DN1549_c0_g1 | 2 |  |
|  |  | TRINITY_DN3511_c0_g1 | 1 |  |
|  | Carbohydrate metabolism | TRINITY_DN1165_c0_g3 | 19 | √ |
|  |  | TRINITY_DN1234_c0_g1 | 19 | √ |
|  |  | TRINITY_DN1257_c0_g2 | 19 | √ |
|  |  | TRINITY_DN1821_c0_g1 | 19 | √ |
|  |  | TRINITY_DN3579_c0_g1 | 19 | √ |
|  |  | TRINITY_DN3779_c0_g1 | 19 | √ |
|  |  | TRINITY_DN4014_c0_g2 | 19 | √ |
|  |  | TRINITY_DN4017_c0_g4 | 19 | √ |
|  |  | TRINITY_DN4136_c0_g1 | 19 | √ |
|  |  | TRINITY_DN4427_c0_g1 | 19 | √ |
|  |  | TRINITY_DN4491_c0_g1 | 19 | √ |
|  |  | TRINITY_DN4625_c0_g3 | 19 | √ |
|  |  | TRINITY_DN4852_c0_g3 | 19 | √ |
|  |  | TRINITY_DN6423_c0_g1 | 19 | √ |
|  |  | TRINITY_DN1445_c0_g1 | 17 |  |
|  |  | TRINITY_DN556_c0_g3 | 17 |  |
|  |  | TRINITY_DN583_c0_g2 | 17 |  |
|  |  | TRINITY_DN785_c0_g1 | 17 |  |
|  |  | TRINITY_DN3050_c0_g3 | 13 |  |
|  |  | TRINITY_DN1871_c0_g1 | 12 |  |
|  |  | TRINITY_DN1391_c0_g1 | 11 |  |
|  |  | TRINITY_DN4170_c0_g4 | 11 |  |
|  |  | TRINITY_DN5859_c0_g1 | 11 |  |
|  |  | TRINITY_DN980_c0_g1 | 11 |  |
|  |  | TRINITY_DN1891_c0_g1 | 11 |  |
|  |  | TRINITY_DN3259_c0_g1 | 11 |  |
|  |  | TRINITY_DN4544_c0_g1 | 11 |  |
|  |  | TRINITY_DN4649_c0_g1 | 11 |  |
|  |  | TRINITY_DN72_c0_g1 | 11 |  |
|  |  | TRINITY_DN750_c0_g1 | 11 |  |
|  |  | TRINITY_DN1703_c0_g1 | 11 |  |
|  |  | TRINITY_DN4199_c0_g2 | 11 |  |
|  |  | TRINITY_DN2592_c0_g1 | 9 |  |
|  |  | TRINITY_DN4385_c0_g1 | 9 |  |
|  |  | TRINITY_DN1598_c1_g3 | 9 |  |
|  |  | TRINITY_DN2597_c0_g4 | 9 |  |
|  |  | TRINITY_DN2771_c0_g1 | 9 |  |
|  |  | TRINITY_DN3960_c0_g4 | 9 |  |
|  |  | TRINITY_DN417_c0_g4 | 9 |  |
|  |  | TRINITY_DN717_c0_g4 | 9 |  |
|  |  | TRINITY_DN717_c0_g5 | 9 |  |
|  |  | TRINITY_DN1270_c0_g1 | 8 |  |
|  |  | TRINITY_DN1301_c0_g4 | 8 |  |
|  |  | TRINITY_DN2730_c0_g1 | 8 |  |
|  |  | TRINITY_DN3348_c0_g3 | 8 |  |
|  |  | TRINITY_DN3511_c0_g1 | 8 |  |
|  |  | TRINITY_DN4170_c0_g3 | 8 |  |
|  |  | TRINITY_DN4746_c0_g1 | 8 |  |
|  |  | TRINITY_DN75_c0_g4 | 8 |  |
|  |  | TRINITY_DN1503_c0_g1 | 8 |  |
|  |  | TRINITY_DN2068_c0_g3 | 8 |  |
|  |  | TRINITY_DN3856_c0_g1 | 8 |  |
|  |  | TRINITY_DN4227_c0_g2 | 8 |  |
|  |  | TRINITY_DN3387_c0_g5 | 6 |  |
|  |  | TRINITY_DN3325_c1_g2 | 6 |  |
|  |  | TRINITY_DN4651_c0_g1 | 6 |  |
|  |  | TRINITY_DN583_c0_g1 | 6 |  |
|  |  | TRINITY_DN6536_c0_g1 | 6 |  |
|  |  | TRINITY_DN2026_c0_g1 | 5 |  |
|  |  | TRINITY_DN2380_c0_g1 | 5 |  |
|  |  | TRINITY_DN2257_c0_g2 | 5 |  |
|  |  | TRINITY_DN1600_c0_g1 | 4 |  |
|  |  | TRINITY_DN5261_c0_g3 | 3 |  |
|  |  | TRINITY_DN2509_c0_g1 | 3 |  |
|  |  | TRINITY_DN2449_c0_g2 | 3 |  |
|  |  | TRINITY_DN2818_c0_g1 | 3 |  |
|  |  | TRINITY_DN3344_c0_g2 | 3 |  |
|  |  | TRINITY_DN2581_c0_g2 | 2 |  |
|  |  | TRINITY_DN1746_c0_g1 | 2 |  |
|  |  | TRINITY_DN886_c0_g4 | 2 |  |
|  |  | TRINITY_DN1145_c0_g2 | 1 |  |
|  |  | TRINITY_DN2291_c0_g1 | 1 |  |
|  | Lipid metabolism | TRINITY_DN2941_c0_g1 | 8 | √ |
|  |  | TRINITY_DN2305_c0_g1 | 7 | √ |
|  |  | TRINITY_DN1687_c0_g2 | 5 | √ |
|  |  | TRINITY_DN6029_c0_g1 | 5 | √ |
|  |  | TRINITY_DN922_c0_g2 | 5 |  |
|  |  | TRINITY_DN964_c0_g5 | 5 | √ |
|  |  | TRINITY_DN2212_c0_g1 | 5 | √ |
|  |  | TRINITY_DN2565_c0_g1 | 5 | √ |
|  |  | TRINITY_DN4491_c0_g1 | 5 | √ |
|  |  | TRINITY_DN860_c0_g3 | 5 |  |
|  |  | TRINITY_DN4651_c0_g1 | 4 |  |
|  |  | TRINITY_DN4471_c0_g1 | 4 |  |
|  |  | TRINITY_DN571_c0_g1 | 4 |  |
|  |  | TRINITY_DN1029_c0_g3 | 3 |  |
|  |  | TRINITY_DN1349_c0_g1 | 3 |  |
|  |  | TRINITY_DN1989_c0_g1 | 3 |  |
|  |  | TRINITY_DN2590_c0_g2 | 3 |  |
|  |  | TRINITY_DN1499_c0_g1 | 3 |  |
|  |  | TRINITY_DN1746_c0_g1 | 3 |  |
|  |  | TRINITY_DN2377_c0_g4 | 3 |  |
|  |  | TRINITY_DN4440_c0_g4 | 3 |  |
|  |  | TRINITY_DN2026_c0_g1 | 3 |  |
|  |  | TRINITY_DN3361_c0_g1 | 3 |  |
|  |  | TRINITY_DN3552_c0_g1 | 3 |  |
|  |  | TRINITY_DN800_c0_g1 | 3 |  |
|  |  | TRINITY_DN3162_c0_g2 | 3 |  |
|  |  | TRINITY_DN3176_c0_g2 | 3 |  |
|  |  | TRINITY_DN2311_c0_g3 | 3 |  |
|  |  | TRINITY_DN2833_c0_g1 | 3 |  |
|  |  | TRINITY_DN5080_c0_g1 | 3 |  |
|  |  | TRINITY_DN4385_c0_g1 | 3 |  |
|  |  | TRINITY_DN5859_c0_g1 | 3 |  |
|  |  | TRINITY_DN742_c0_g2 | 3 |  |
|  |  | TRINITY_DN1171_c0_g1 | 2 |  |
|  |  | TRINITY_DN3856_c0_g1 | 2 |  |
|  |  | TRINITY_DN252_c0_g1 | 2 |  |
|  |  | TRINITY_DN447_c1_g1 | 2 |  |
|  |  | TRINITY_DN1134_c0_g1 | 1 |  |
|  |  | TRINITY_DN6759_c0_g1 | 1 |  |
|  |  | TRINITY_DN2570_c0_g1 | 1 |  |
|  |  | TRINITY_DN2797_c0_g1 | 1 |  |
|  |  | TRINITY_DN939_c0_g1 | 1 |  |
|  | Amino acid metabolism | TRINITY_DN1363_c0_g1 | 12 | √ |
|  |  | TRINITY_DN327_c0_g1 | 12 | √ |
|  |  | TRINITY_DN6810_c0_g1 | 12 | √ |
|  |  | TRINITY_DN3260_c0_g1 | 10 | √ |
|  |  | TRINITY_DN4491_c0_g1 | 10 | √ |
|  |  | TRINITY_DN78_c0_g1 | 10 | √ |
|  |  | TRINITY_DN1058_c0_g1 | 9 | √ |
|  |  | TRINITY_DN2178_c0_g1 | 9 | √ |
|  |  | TRINITY_DN432_c0_g5 | 9 | √ |
|  |  | TRINITY_DN72_c0_g1 | 9 | √ |
|  |  | TRINITY_DN2382_c0_g3 | 9 |  |
|  |  | TRINITY_DN4640_c0_g1 | 9 |  |
|  |  | TRINITY_DN5840_c0_g1 | 9 |  |
|  |  | TRINITY_DN3856_c0_g1 | 7 |  |
|  |  | TRINITY_DN3555_c0_g3 | 7 |  |
|  |  | TRINITY_DN1781_c0_g1 | 6 |  |
|  |  | TRINITY_DN4627_c0_g1 | 6 |  |
|  |  | TRINITY_DN1197_c0_g1 | 6 |  |
|  |  | TRINITY_DN2538_c0_g1 | 6 |  |
|  |  | TRINITY_DN5859_c0_g1 | 6 |  |
|  |  | TRINITY_DN980_c0_g1 | 6 |  |
|  |  | TRINITY_DN1891_c0_g1 | 6 |  |
|  |  | TRINITY_DN4544_c0_g1 | 6 |  |
|  |  | TRINITY_DN2026_c0_g1 | 6 |  |
|  |  | TRINITY_DN259_c0_g1 | 6 |  |
|  |  | TRINITY_DN337_c0_g1 | 6 |  |
|  |  | TRINITY_DN3301_c0_g1 | 5 |  |
|  |  | TRINITY_DN3301_c0_g2 | 5 |  |
|  |  | TRINITY_DN1908_c0_g2 | 5 |  |
|  |  | TRINITY_DN1630_c0_g1 | 4 |  |
|  |  | TRINITY_DN3428_c0_g1 | 4 |  |
|  |  | TRINITY_DN3466_c0_g1 | 4 |  |
|  |  | TRINITY_DN3955_c0_g1 | 4 |  |
|  |  | TRINITY_DN1082_c0_g1 | 3 |  |
|  |  | TRINITY_DN2088_c0_g5 | 3 |  |
|  |  | TRINITY_DN2709_c0_g1 | 3 |  |
|  |  | TRINITY_DN3955_c0_g2 | 3 |  |
|  |  | TRINITY_DN1391_c0_g3 | 3 |  |
|  |  | TRINITY_DN1666_c0_g1 | 3 |  |
|  |  | TRINITY_DN3827_c0_g1 | 3 |  |
|  |  | TRINITY_DN4640_c0_g2 | 3 |  |
|  |  | TRINITY_DN4385_c0_g1 | 3 |  |
|  |  | TRINITY_DN1145_c0_g2 | 2 |  |
|  |  | TRINITY_DN5916_c0_g2 | 2 |  |
|  |  | TRINITY_DN835_c0_g1 | 2 |  |
|  |  | TRINITY_DN2380_c0_g1 | 2 |  |
|  |  | TRINITY_DN3500_c0_g1 | 2 |  |
|  |  | TRINITY_DN409_c0_g3 | 2 |  |
|  |  | TRINITY_DN6209_c0_g1 | 1 |  |
| Cellular Processes | Cell growth and death | TRINITY_DN1583_c0_g1 | 12 | √ |
|  |  | TRINITY_DN1643_c0_g1 | 12 |  |
|  |  | TRINITY_DN3552_c0_g3 | 12 | √ |
|  |  | TRINITY_DN409_c0_g5 | 12 |  |
|  |  | TRINITY_DN4750_c0_g3 | 12 | √ |
|  |  | TRINITY_DN507_c0_g1 | 12 | √ |
|  |  | TRINITY_DN5563_c0_g1 | 12 | √ |
|  |  | TRINITY_DN988_c0_g2 | 12 |  |
|  |  | TRINITY_DN2322_c0_g1 | 10 |  |
|  |  | TRINITY_DN5563_c0_g2 | 10 |  |
|  |  | TRINITY_DN5771_c0_g1 | 10 |  |
|  |  | TRINITY_DN6304_c0_g1 | 10 |  |
|  |  | TRINITY_DN4530_c0_g1 | 8 |  |
|  |  | TRINITY_DN3384_c0_g1 | 3 |  |
|  |  | TRINITY_DN1236_c0_g3 | 2 |  |
|  |  | TRINITY_DN3325_c1_g4 | 2 |  |
|  |  | TRINITY_DN1994_c0_g1 | 2 |  |
|  |  | TRINITY_DN3481_c0_g4 | 2 |  |
|  |  | TRINITY_DN3204_c0_g1 | 2 |  |
|  |  | TRINITY_DN1976_c0_g1 | 1 |  |
|  |  | TRINITY_DN2056_c0_g1 | 1 |  |
|  |  | TRINITY_DN2071_c0_g4 | 1 |  |
|  |  | TRINITY_DN4730_c0_g1 | 1 |  |
|  |  | TRINITY_DN618_c0_g2 | 1 |  |
|  | Transport and catabolism | TRINITY_DN1906_c0_g1 | 13 |  |
|  |  | TRINITY_DN1967_c0_g2 | 13 | √ |
|  |  | TRINITY_DN2549_c0_g1 | 13 | √ |
|  |  | TRINITY_DN28_c0_g2 | 13 | √ |
|  |  | TRINITY_DN410_c0_g2 | 13 | √ |
|  |  | TRINITY_DN953_c0_g1 | 13 | √ |
|  |  | TRINITY_DN1029_c0_g3 | 10 | √ |
|  |  | TRINITY_DN2076_c0_g2 | 10 | √ |
|  |  | TRINITY_DN3007_c0_g1 | 10 | √ |
|  |  | TRINITY_DN3198_c0_g1 | 10 |  |
|  |  | TRINITY_DN699_c0_g1 | 10 | √ |
|  |  | TRINITY_DN72_c0_g1 | 10 |  |
|  |  | TRINITY_DN1022_c0_g2 | 9 |  |
|  |  | TRINITY_DN2071_c0_g4 | 9 |  |
|  |  | TRINITY_DN2257_c0_g2 | 9 |  |
|  |  | TRINITY_DN2771_c0_g1 | 9 |  |
|  |  | TRINITY_DN2785_c0_g2 | 9 |  |
|  |  | TRINITY_DN6000_c0_g1 | 9 |  |
|  |  | TRINITY_DN751_c0_g1 | 9 |  |
|  |  | TRINITY_DN1891_c0_g1 | 8 |  |
|  |  | TRINITY_DN4750_c0_g4 | 8 |  |
|  |  | TRINITY_DN3535_c0_g2 | 7 |  |
|  |  | TRINITY_DN417_c0_g4 | 7 |  |
|  |  | TRINITY_DN2161_c0_g1 | 7 |  |
|  |  | TRINITY_DN49_c0_g1 | 6 |  |
|  |  | TRINITY_DN1732_c0_g2 | 5 |  |
|  |  | TRINITY_DN3636_c0_g1 | 5 |  |
|  |  | TRINITY_DN1717_c0_g1 | 5 |  |
|  |  | TRINITY_DN3080_c0_g1 | 5 |  |
|  |  | TRINITY_DN4255_c0_g2 | 5 |  |
|  |  | TRINITY_DN3146_c0_g2 | 5 |  |
|  |  | TRINITY_DN1721_c0_g2 | 4 |  |
|  |  | TRINITY_DN571_c0_g1 | 4 |  |
|  |  | TRINITY_DN6217_c1_g1 | 4 |  |
|  |  | TRINITY_DN796_c0_g2 | 4 |  |
|  |  | TRINITY_DN939_c0_g1 | 4 |  |
|  |  | TRINITY_DN215_c0_g3 | 4 |  |
|  |  | TRINITY_DN3256_c0_g1 | 4 |  |
|  |  | TRINITY_DN4255_c0_g3 | 4 |  |
|  |  | TRINITY_DN158_c0_g3 | 3 |  |
|  |  | TRINITY_DN742_c0_g2 | 3 |  |
|  |  | TRINITY_DN4916_c0_g1 | 3 |  |
|  |  | TRINITY_DN4177_c0_g1 | 3 |  |
|  |  | TRINITY_DN900_c0_g5 | 3 |  |
|  |  | TRINITY_DN1086_c0_g1 | 1 |  |
|  |  | TRINITY_DN21_c0_g1 | 1 |  |
|  |  | TRINITY_DN2476_c0_g1 | 1 |  |
| Genetic Information Processing | Replication and repair | TRINITY_DN1709_c0_g2 | 28 | √ |
|  |  | TRINITY_DN3065_c0_g2 | 28 | √ |
|  |  | TRINITY_DN3987_c0_g1 | 28 | √ |
|  |  | TRINITY_DN5608_c1_g2 | 28 | √ |
|  |  | TRINITY_DN5808_c0_g4 | 28 |  |
|  |  | TRINITY_DN6036_c0_g1 | 28 |  |
|  |  | TRINITY_DN6304_c0_g1 | 28 |  |
|  |  | TRINITY_DN6583_c0_g1 | 28 | √ |
|  |  | TRINITY_DN6819_c0_g1 | 28 | √ |
|  |  | TRINITY_DN5798_c0_g1 | 27 |  |
|  |  | TRINITY_DN1090_c0_g3 | 26 |  |
|  |  | TRINITY_DN1354_c0_g6 | 26 |  |
|  |  | TRINITY_DN283_c0_g1 | 26 |  |
|  |  | TRINITY_DN2995_c0_g1 | 26 |  |
|  |  | TRINITY_DN327_c0_g1 | 26 |  |
|  |  | TRINITY_DN3747_c0_g1 | 26 |  |
|  |  | TRINITY_DN409_c0_g5 | 26 |  |
|  |  | TRINITY_DN4754_c0_g1 | 26 |  |
|  |  | TRINITY_DN5190_c2_g1 | 26 |  |
|  |  | TRINITY_DN6151_c0_g1 | 26 |  |
|  |  | TRINITY_DN988_c0_g2 | 26 |  |
|  |  | TRINITY_DN1453_c0_g1 | 25 |  |
|  |  | TRINITY_DN2322_c0_g1 | 25 |  |
|  |  | TRINITY_DN4650_c0_g1 | 25 |  |
|  |  | TRINITY_DN5618_c0_g1 | 25 |  |
|  |  | TRINITY_DN6650_c0_g1 | 25 |  |
|  |  | TRINITY_DN3476_c0_g2 | 22 |  |
|  |  | TRINITY_DN1885_c0_g5 | 12 |  |
|  |  | TRINITY_DN2872_c0_g3 | 11 |  |
|  |  | TRINITY_DN1722_c0_g1 | 1 |  |
|  |  | TRINITY_DN2722_c0_g1 | 1 |  |
|  |  | TRINITY_DN3186_c0_g1 | 1 |  |
|  | Translation | TRINITY_DN1610_c0_g3 | 20 |  |
|  |  | TRINITY_DN2100_c0_g3 | 20 |  |
|  |  | TRINITY_DN2257_c0_g2 | 20 | √ |
|  |  | TRINITY_DN3118_c0_g5 | 20 |  |
|  |  | TRINITY_DN4630_c0_g2 | 20 | √ |
|  |  | TRINITY_DN5782_c0_g1 | 20 | √ |
|  |  | TRINITY_DN6555_c0_g1 | 20 | √ |
|  |  | TRINITY_DN2044_c0_g2 | 16 | √ |
|  |  | TRINITY_DN2479_c0_g2 | 16 | √ |
|  |  | TRINITY_DN2802_c0_g2 | 16 | √ |
|  |  | TRINITY_DN2916_c0_g1 | 16 | √ |
|  |  | TRINITY_DN428_c0_g1 | 16 | √ |
|  |  | TRINITY_DN46_c0_g2 | 16 | √ |
|  |  | TRINITY_DN659_c0_g1 | 16 | √ |
|  |  | TRINITY_DN1969_c0_g4 | 14 |  |
|  |  | TRINITY_DN3480_c0_g3 | 14 |  |
|  |  | TRINITY_DN379_c0_g1 | 14 |  |
|  |  | TRINITY_DN4215_c0_g1 | 14 |  |
|  |  | TRINITY_DN677_c1_g1 | 14 |  |
|  |  | TRINITY_DN1384_c0_g3 | 10 |  |
|  |  | TRINITY_DN2736_c1_g2 | 10 |  |
|  |  | TRINITY_DN3278_c1_g2 | 10 |  |
|  |  | TRINITY_DN4255_c0_g4 | 10 |  |
|  |  | TRINITY_DN5862_c0_g2 | 10 |  |
|  |  | TRINITY_DN1154_c0_g2 | 9 |  |
|  |  | TRINITY_DN3447_c0_g1 | 9 |  |
|  |  | TRINITY_DN514_c0_g3 | 9 |  |
|  |  | TRINITY_DN5613_c0_g1 | 9 |  |
|  |  | TRINITY_DN2300_c0_g1 | 8 |  |
|  |  | TRINITY_DN3061_c0_g2 | 8 |  |
|  |  | TRINITY_DN3867_c0_g4 | 8 |  |
|  |  | TRINITY_DN132_c0_g3 | 8 |  |
|  |  | TRINITY_DN2340_c0_g1 | 8 |  |
|  |  | TRINITY_DN5866_c0_g1 | 8 |  |
|  |  | TRINITY_DN2642_c1_g1 | 8 |  |
|  |  | TRINITY_DN288_c0_g1 | 6 |  |
|  |  | TRINITY_DN1915_c0_g2 | 6 |  |
|  |  | TRINITY_DN419_c0_g1 | 6 |  |
|  |  | TRINITY_DN2127_c0_g2 | 5 |  |
|  |  | TRINITY_DN215_c0_g3 | 5 |  |
|  |  | TRINITY_DN3443_c0_g1 | 5 |  |
|  |  | TRINITY_DN891_c0_g1 | 5 |  |
|  |  | TRINITY_DN3421_c0_g5 | 5 |  |
|  |  | TRINITY_DN3187_c0_g1 | 5 |  |
|  |  | TRINITY_DN1218_c0_g1 | 3 |  |
|  |  | TRINITY_DN2617_c0_g2 | 3 |  |
|  |  | TRINITY_DN3668_c0_g2 | 3 |  |
|  |  | TRINITY_DN168_c0_g1 | 2 |  |
|  |  | TRINITY_DN2320_c0_g1 | 2 |  |
|  |  | TRINITY_DN3212_c0_g1 | 2 |  |
|  |  | TRINITY_DN981_c0_g2 | 2 |  |
|  |  | TRINITY_DN3829_c0_g1 | 2 |  |
|  |  | TRINITY_DN506_c0_g1 | 2 |  |
|  |  | TRINITY_DN4892_c0_g2 | 1 |  |
|  | Transcription | TRINITY_DN1833_c0_g2 | 15 | √ |
|  |  | TRINITY_DN2374_c0_g1 | 15 | √ |
|  |  | TRINITY_DN4133_c0_g2 | 15 | √ |
|  |  | TRINITY_DN1963_c0_g2 | 11 | √ |
|  |  | TRINITY_DN2249_c0_g1 | 11 | √ |
|  |  | TRINITY_DN3668_c0_g3 | 11 |  |
|  |  | TRINITY_DN3843_c0_g1 | 11 | √ |
|  |  | TRINITY_DN3993_c0_g2 | 11 |  |
|  |  | TRINITY_DN4097_c0_g1 | 11 |  |
|  |  | TRINITY_DN3221_c0_g1 | 9 |  |
|  |  | TRINITY_DN3811_c0_g2 | 9 |  |
|  |  | TRINITY_DN4080_c0_g3 | 9 |  |
|  |  | TRINITY_DN4433_c1_g3 | 9 |  |
|  |  | TRINITY_DN4581_c0_g3 | 9 |  |
|  |  | TRINITY_DN1361_c0_g1 | 8 |  |
|  |  | TRINITY_DN176_c0_g2 | 8 |  |
|  |  | TRINITY_DN3825_c0_g2 | 7 |  |
|  |  | TRINITY_DN4650_c0_g1 | 6 |  |
|  |  | TRINITY_DN529_c0_g1 | 6 |  |
|  |  | TRINITY_DN5030_c0_g4 | 6 |  |
|  |  | TRINITY_DN1154_c0_g2 | 5 |  |
|  |  | TRINITY_DN1967_c0_g2 | 5 |  |
|  |  | TRINITY_DN2929_c0_g1 | 5 |  |
|  |  | TRINITY_DN2642_c1_g1 | 5 |  |
|  |  | TRINITY_DN5542_c0_g2 | 5 |  |
|  |  | TRINITY_DN3941_c0_g3 | 4 |  |
|  |  | TRINITY_DN3139_c0_g5 | 3 |  |
|  |  | TRINITY_DN168_c0_g1 | 2 |  |
|  |  | TRINITY_DN1919_c0_g1 | 2 |  |
|  |  | TRINITY_DN3186_c0_g1 | 1 |  |
|  |  | TRINITY_DN623_c0_g2 | 1 |  |
|  |  | TRINITY_DN3698_c0_g1 | 1 |  |
|  | Folding, sorting and degradation | TRINITY_DN216_c0_g1 | 9 | √ |
|  |  | TRINITY_DN846_c0_g1 | 8 | √ |
|  |  | TRINITY_DN2501_c0_g1 | 6 | √ |
|  |  | TRINITY_DN529_c0_g1 | 6 | √ |
|  |  | TRINITY_DN1121_c0_g2 | 5 |  |
|  |  | TRINITY_DN189_c0_g2 | 5 | √ |
|  |  | TRINITY_DN3139_c0_g5 | 5 |  |
|  |  | TRINITY_DN3214_c0_g1 | 5 |  |
|  |  | TRINITY_DN3242_c1_g1 | 5 |  |
|  |  | TRINITY_DN348_c0_g1 | 5 |  |
|  |  | TRINITY_DN4441_c1_g3 | 5 |  |
|  |  | TRINITY_DN4750_c0_g3 | 5 |  |
|  |  | TRINITY_DN5340_c0_g6 | 5 |  |
|  |  | TRINITY_DN6536_c0_g1 | 5 |  |
|  |  | TRINITY_DN1598_c1_g1 | 4 |  |
|  |  | TRINITY_DN1774_c0_g3 | 4 |  |
|  |  | TRINITY_DN2059_c0_g1 | 4 |  |
|  |  | TRINITY_DN2738_c0_g2 | 4 |  |
|  |  | TRINITY_DN301_c0_g1 | 4 |  |
|  |  | TRINITY_DN3786_c0_g4 | 4 |  |
|  |  | TRINITY_DN4441_c1_g1 | 4 |  |
|  |  | TRINITY_DN4730_c0_g1 | 4 |  |
|  |  | TRINITY_DN900_c0_g5 | 4 |  |
|  |  | TRINITY_DN1121_c0_g1 | 3 |  |
|  |  | TRINITY_DN1588_c0_g3 | 3 |  |
|  |  | TRINITY_DN2716_c0_g1 | 3 |  |
|  |  | TRINITY_DN286_c0_g1 | 3 |  |
|  |  | TRINITY_DN3118_c0_g3 | 3 |  |
|  |  | TRINITY_DN3481_c0_g4 | 3 |  |
|  |  | TRINITY_DN4253_c0_g1 | 3 |  |
|  |  | TRINITY_DN4554_c0_g4 | 3 |  |
|  |  | TRINITY_DN618_c0_g1 | 3 |  |
|  |  | TRINITY_DN618_c0_g2 | 3 |  |
|  |  | TRINITY_DN1522_c0_g2 | 2 |  |
|  |  | TRINITY_DN1610_c0_g1 | 2 |  |
|  |  | TRINITY_DN2749_c0_g1 | 2 |  |
|  |  | TRINITY_DN4667_c0_g1 | 2 |  |
|  |  | TRINITY_DN5253_c0_g1 | 2 |  |
|  |  | TRINITY_DN5335_c0_g2 | 2 |  |
|  |  | TRINITY_DN897_c0_g3 | 2 |  |
|  |  | TRINITY_DN1885_c0_g2 | 1 |  |

Table S3. Hub genes and target proteins.

| Hub genes | Protein PDB | Resolution (Ǻ) |
| --- | --- | --- |
| TRINITY_DN6823_c0_g1 | 1QLE | 3.00 |
| TRINITY_DN4502_c0_g2 | 1M56 | 2.30 |
| TRINITY_DN6570_c0_g1 | 2CUA | 1.60 |
| TRINITY_DN5144_c0_g1 | 6YNW | 3.10 |
| TRINITY_DN5144_c0_g2 | 5Z62 | 3.60 |
| TRINITY_DN6120_c0_g1 | 6ADQ | 3.50 |
| TRINITY_DN1165_c0_g3 | 3DR2 | 1.67 |
| TRINITY_DN1234_c0_g1 | 2GQ1 | 1.45 |
| TRINITY_DN1257_c0_g2 | 1FUO | 1.98 |
| TRINITY_DN1821_c0_g1 | 1BDG | 2.60 |
| TRINITY_DN3579_c0_g1 | 7STL | 2.95 |
| TRINITY_DN3779_c0_g1 | 2WSK | 2.25 |
| TRINITY_DN4014_c0_g2 | 3CMJ | 1.60 |
| TRINITY_DN4017_c0_g4 | 2QZS | 2.20 |
| TRINITY_DN4136_c0_g1 | 1M7X | 2.30 |
| TRINITY_DN4427_c0_g1 | 1E4I | 2.00 |
| TRINITY_DN4491_c0_g1 | 4GKV | 2.01 |
| TRINITY_DN4625_c0_g3 | 1FCB | 2.40 |
| TRINITY_DN4852_c0_g3 | 6AGS | 2.31 |
| TRINITY_DN6423_c0_g1 | 6JAK | 2.41 |
| TRINITY_DN2941_c0_g1 | 1BCI | - |
| TRINITY_DN2305_c0_g1 | 1BWP | 2.10 |
| TRINITY_DN1687_c0_g2 | 4ZYO | 3.25 |
| TRINITY_DN6029_c0_g1 | 6C6N | 2.30 |
| TRINITY_DN964_c0_g5 | 7EWT | 3.40 |
| TRINITY_DN2212_c0_g1 | 4RPE | 1.60 |
| TRINITY_DN2565_c0_g1 | 3VZB | 2.00 |
| TRINITY_DN4491_c0_g1 | 4GKV | 2.01 |
| TRINITY_DN1363_c0_g1 | 4MI0 | 2.00 |
| TRINITY_DN327_c0_g1 | 2UXY | 1.25 |
| TRINITY_DN6810_c0_g1 | 6IG5 | 2.08 |
| TRINITY_DN3260_c0_g1 | 4AQ6 | 1.98 |
| TRINITY_DN4491_c0_g1 | 4GKV | 2.01 |
| TRINITY_DN78_c0_g1 | 7SF5 | 2.52 |
| TRINITY_DN1058_c0_g1 | 1YFU | 1.90 |
| TRINITY_DN2178_c0_g1 | 2CTZ | 2.60 |
| TRINITY_DN432_c0_g5 | 1SC6 | 2.09 |
| TRINITY_DN72_c0_g1 | 1GWE | 0.88 |
| TRINITY_DN1583_c0_g1 | 5XW4 | 1.85 |
| TRINITY_DN3552_c0_g3 | 1ZLT | 1.74 |
| TRINITY_DN4750_c0_g3 | 4GGA | 2.04 |
| TRINITY_DN507_c0_g1 | 6WG4 | 2.31 |
| TRINITY_DN5563_c0_g1 | 6IGX | 3.00 |
| TRINITY_DN1967_c0_g2 | 4JSN | 3.20 |
| TRINITY_DN2549_c0_g1 | 2FA2 | 2.85 |
| TRINITY_DN28_c0_g2 | 1PL4 | 1.47 |
| TRINITY_DN410_c0_g2 | 3RUJ | 2.10 |
| TRINITY_DN953_c0_g1 | 4BX8 | 2.40 |
| TRINITY_DN1029_c0_g3 | 7K0I | 3.30 |
| TRINITY_DN2076_c0_g2 | 2ZME | 2.90 |
| TRINITY_DN3007_c0_g1 | 3GGZ | 3.80 |
| TRINITY_DN699_c0_g1 | 6RGO | 3.70 |
| TRINITY_DN1709_c0_g2 | 6WVK | 3.36 |
| TRINITY_DN3065_c0_g2 | 1GS0 | 2.80 |
| TRINITY_DN3987_c0_g1 | 3THW | 3.09 |
| TRINITY_DN5608_c1_g2 | 6DI2 | 1.32 |
| TRINITY_DN6583_c0_g1 | 7STE | 2.73 |
| TRINITY_DN6819_c0_g1 | 3L0X | 3.00 |
| TRINITY_DN2257_c0_g2 | 5GZT | 2.10 |
| TRINITY_DN4630_c0_g2 | 2JVO | - |
| TRINITY_DN5782_c0_g1 | 7APK | 3.30 |
| TRINITY_DN2044_c0_g2 | 3EGI | 2.21 |
| TRINITY_DN2479_c0_g2 | 7EIV | 2.68 |
| TRINITY_DN2802_c0_g2 | 2C47 | 2.40 |
| TRINITY_DN2916_c0_g1 | 6ZCE | 5.30 |
| TRINITY_DN428_c0_g1 | 6EOJ | 3.55 |
| TRINITY_DN46_c0_g2 | 4YXP | 1.92 |
| TRINITY_DN659_c0_g1 | 5MTZ | 2.99 |
| TRINITY_DN1833_c0_g2 | 5LSB | 2.70 |
| TRINITY_DN2374_c0_g1 | 2XAU | 1.90 |
| TRINITY_DN4133_c0_g2 | 5LJ5 | 10.00 |
| TRINITY_DN1963_c0_g2 | 2LO6 | - |
| TRINITY_DN2249_c0_g1 | 4PYU | 2.00 |
| TRINITY_DN3843_c0_g1 | 5LMX | 4.90 |
| TRINITY_DN216_c0_g1 | 4RZK | 2.68 |
| TRINITY_DN846_c0_g1 | 4BUJ | 3.70 |
| TRINITY_DN2501_c0_g1 | 2QGX | 2.56 |
| TRINITY_DN529_c0_g1 | 5M8C | 2.30 |
| TRINITY_DN189_c0_g2 | 1KKT | 2.20 |

Table S4. Hydrogen bonds existence rate analysis during MDS.

| A5-4ZYO | | A11-4ZYO | | A30-4ZYO | | B74-5XW4 | | B75-1FCB | | B63-7O3C | |
| --- | --- | --- | --- | --- | --- | --- | --- | --- | --- | --- | --- |
| Donnor-Acceptor | Rate (%) | Donnor-Acceptor | Rate (%) | Donnor-Acceptor | Rate (%) | Donnor-Acceptor | Rate (%) | Donnor-Acceptor | Rate (%) | Donnor-Acceptor | Rate (%) |
| A5‧‧‧Thr261 | 54.09 | His157‧‧‧A11 | 92.16 | Hsd157‧‧‧A30 | 98.44 | Val180‧‧‧B74 | 94.27 | B75‧‧‧Val70 | 84.73 | B63‧‧‧Leu250 | 24.30 |
| His157‧‧‧A5 | 45.45 | A11‧‧‧Ala292 | 21.04 | Trp262‧‧‧A30 | 49.05 | Asn179‧‧‧B74 | 34.57 | B75‧‧‧Hsd43 | 5.47 | Hsd182‧‧‧B63 | 20.99 |
| Trp153‧‧‧A5 | 38.15 | Trp153‧‧‧A11 | 17.90 | A30‧‧‧Thr261 | 37.58 | Asp158‧‧‧B74 | 13.71 | B75‧‧‧Hsd66 | 4.21 | Tyr273‧‧‧B63 | 6.17 |
| His298‧‧‧A5 | 19.28 | Hsd120‧‧‧A11 | 9.47 | A30‧‧‧Thr261 | 16.58 | Asn163‧‧‧B74 | 5.55 | Hsd66‧‧‧B75 | 3.44 |  |  |
| A5‧‧‧Hsd160 | 17.45 | A11‧‧‧Thr116 | 7.86 | Asn265‧‧‧A30 | 13.69 | B74‧‧‧Ser166 | 5.46 |  |  |  |  |
| A5‧‧‧Glu295 | 12.74 | A11‧‧‧Hsd157 | 6.64 | Gln147‧‧‧A30 | 10.86 |  |  |  |  |  |  |
|  |  | A11‧‧‧Trp153 | 6.36 |  |  |  |  |  |  |  |  |
|  |  | Gln147‧‧‧A11 | 5.16 |  |  |  |  |  |  |  |  |

Table S5. Sequence of target proteins used in RT-qPCR analysis.

| Primer | Sequence (5'to3') | Product (bp) |
| --- | --- | --- |
| 1FCB-F | GCGTGAGATCCTTATGGGCA | 105 |
| 1FCB-R | AGCTTCGGTGCTATGGGTTC |  |
| 1GWE-F | TTTGGTCACGGCAAGGCTAT | 237 |
| 1GWE-R | GTGCTTGCTGACTTCCAACG |  |
| 3CMJ-F | GCGGATGTCGATTTGAAGCC | 123 |
| 3CMJ-R | AAACGTAATCTGGCCCTCCG |  |
| 5XW4-F | GGAGGAGTTTAGTGGTCCGC | 189 |
| 5XW4-R | CATCTTCTCCTCCCTGCCTG |  |
| 6C6N-F | ATCTGGTAGAGGAGGACGGG | 171 |
| 6C6N-R | CGACGGATGCTTCTCCAACT |  |

Table S6. Hub genes and target proteins of *Escherichia* *coli* treated with *C*. *camphora* oil.

| KEGG first categories | KEGG second categories | Hub gene | Degree | PDB ID |
| --- | --- | --- | --- | --- |
| Metabolism | Carbohydrate metabolism | b0114 | 136 | 2G67 |
|  |  | b0115 | 136 | 7B9K |
|  |  | b3011 | 136 | 1OJ7 |
|  |  | b4383 | 136 | 3M7V |
|  |  | b0755 | 134 | 1E58 |
|  |  | b0903 | 134 | 1H16 |
|  |  | b0927 | 134 | 2QED |
|  |  | b1779 | 134 | 6IO4 |
|  |  | b3255 | 134 | 4HR7 |
|  |  | b3925 | 134 | 3D1R |
|  |  | b4197 | 134 | 3CQH |
|  |  | b0116 | 132 | 4JDR |
|  |  | b1611 | 132 | 1KQ7 |
|  |  | b2388 | 132 | 1Q18 |
|  |  | b3752 | 132 | 1RKS |
|  |  | b4196 | 132 | 1SO3 |
|  |  | b4322 | 130 | 4EAC |
|  |  | b2247 | 128 | 2I5Q |
|  |  | b2463 | 128 | 6ZN7 |
|  |  | b3236 | 128 | 1IB6 |
|  |  | b2715 | 126 | 1O2F |
|  |  | b0727 | 124 | 1C4T |
|  |  | b1617 | 124 | 3K4A |
|  | Amino acid metabolism | b0116 | 88 | 4JDR |
|  |  | b0159 | 88 | 4WKC |
|  |  | b0242 | 88 | 2J5V |
|  |  | b0311 | 88 | 3Q9T |
|  |  | b0754 | 88 | 1KFL |
|  |  | b0755 | 88 | 1E58 |
|  |  | b1189 | 88 | 5GZ3 |
|  |  | b1622 | 88 | 1D2F |
|  |  | b2021 | 88 | 1GEX |
|  |  | b2290 | 88 | 4CVQ |
|  |  | b2414 | 88 | 5J43 |
|  |  | b2472 | 88 | 7T1Q |
|  |  | b3213 | 88 | 1EA0 |
|  |  | b3236 | 88 | 1IB6 |
|  |  | b3433 | 88 | 1T4B |
|  |  | b3774 | 88 | 1YRL |
|  | Energy metabolism | b0429 | 102 | 7CUB |
|  |  | b0430 | 102 | 7CUB |
|  |  | b0729 | 102 | 2SCU |
|  |  | b0755 | 102 | 1E58 |
|  |  | b0873 | 102 | 7DE4 |
|  |  | b1136 | 102 | 1IDF |
|  |  | b1225 | 102 | 1Q16 |
|  |  | b1611 | 102 | 6NZB |
|  |  | b1779 | 102 | 1DC3 |
|  |  | b2276 | 102 | 7NYH |
|  |  | b2277 | 102 | 7NYH |
|  |  | b2280 | 102 | 7NYH |
|  |  | b2414 | 102 | 5J43 |
|  | Metabolism of cofactors and vitamins | b0369 | 90 | 1I8J |
|  |  | b0415 | 90 | 1KYY |
|  |  | b0630 | 90 | 2QHT |
|  |  | b1288 | 90 | 1DFG |
|  |  | b1638 | 90 | 1JNW |
|  |  | b2260 | 90 | 5C5H |
|  |  | b2315 | 90 | 1W78 |
|  |  | b2530 | 90 | 1P3W |
|  |  | b4211 | 90 | 2ZCV |
|  |  | b4384 | 90 | 1ECP |
|  |  | b0133 | 88 | 1IHO |
|  |  | b0414 | 88 | 2G6V |
|  |  | b0781 | 88 | 1MKZ |
|  | Nucleotide metabolism | b0523 | 56 | 1D7A |
|  |  | b3806 | 54 | 3G82 |
|  |  | b1849 | 52 | 1EYZ |
|  |  | b2312 | 52 | 1ECF |
|  |  | b2476 | 52 | 2GQR |
|  |  | b2499 | 52 | 1CLI |
|  |  | b2508 | 52 | 2CU0 |
|  |  | b4006 | 52 | 1G8M |
|  |  | b4238 | 52 | 1H78 |
|  |  | b0032 | 50 | 1BXR |
|  |  | b2518 | 50 | 2HUR |
| Environmental information processing | Membrane transport | b2415 | 76 | 3EZA |
|  |  | b3195 | 76 | 6XGZ |
|  |  | b3204 | 76 | 1A6J |
|  |  | b3981 | 76 | 5MG3 |
|  |  | b1116 | 74 | 5NAA |
|  |  | b2715 | 74 | 1O2F |
|  |  | b3193 | 74 | 5UW2 |
|  |  | b3194 | 74 | 7CH7 |
|  |  | b2416 | 72 | 1EZA |
|  |  | b1117 | 66 | 7ARI |
|  |  | b3751 | 66 | 1URP |
|  |  | b0098 | 64 | 2FSF |
|  |  | b3192 | 64 | 5UWA |
|  |  | b0408 | 62 | 5MG3 |
|  |  | b3201 | 62 | 4P32 |
|  |  | b1709 | 60 | 1L7V |
|  | Signal transport | b2518 | 92 | 2HUR |
|  |  | b1887 | 90 | 2HO9 |
|  |  | b2994 | 90 | 6EHQ |
|  |  | b3357 | 90 | 3QOP |
|  |  | b4152 | 88 | 1KF6 |
|  |  | b4401 | 86 | 1XHE |
|  |  | b0929 | 84 | 4GCP |
|  |  | b1886 | 84 | 2ASR |
|  |  | b1922 | 84 | 6PMJ |
|  |  | b2253 | 84 | 8SNJ |
|  |  | b2997 | 84 | 6EHQ |
|  |  | b3072 | 84 | 8DIK |
|  |  | b4153 | 84 | 1KF6 |
|  |  | b2369 | 84 | 3F6C |
|  |  | b4151 | 82 | 1KF6 |
| Genetic information processing | Replication and repair | b0467 | 46 | 2RT6 |
|  |  | b0470 | 46 | 1JR3 |
|  |  | b0472 | 46 | 1HJR |
|  |  | b1749 | 46 | 1AKO |
|  |  | b1844 | 46 | 4FZX |
|  |  | b1863 | 46 | 1HJR |
|  | Translation | b1637 | 38 | 1VBM |
|  |  | b1714 | 38 | 6P24 |
|  |  | b1716 | 38 | 3BBX |
|  |  | b1719 | 38 | 1EVK |
|  | Folding, sorting and degration | b0782 | 32 | 1MKZ |
|  |  | b0784 | 32 | 1FM0 |
|  |  | b1133 | 32 | 2DER |
| Cellutar processes | Cellular community | b3357 | 74 | 2GZW |
|  |  | b3517 | 74 | 1XEY |
|  |  | b1922 | 72 | 6PMJ |
|  |  | b4401 | 72 | 1XHE |
|  |  | b1441 | 72 | 1Z47 |
|  |  | b1805 | 72 | 3G7S |
|  |  | b2696 | 72 | 5Z38 |
|  |  | b3417 | 72 | 2ECP |
|  |  | b3533 | 72 | 7LBY |
|  |  | b1194 | 70 | 5Y6F |
|  | Cell motility | b1887 | 48 | 2HO9 |
|  |  | b1886 | 44 | 2ASR |
|  |  | b1888 | 44 | 1A0O |
|  |  | b1922 | 44 | 6PMJ |
|  |  | b1924 | 44 | 5H5V |
| Human disease | Drug resistance: antimicrobial | b0929 | 22 | 2OMF |
|  |  | b2253 | 22 | 8SNJ |
|  |  | b2255 | 22 | 1U9J |
|  |  | b2257 | 22 | 5EZM |
|  |  | b2259 | 22 | 4HN7 |

Table S7. Components in *C*. *camphora* essential oil.

| Number | Substances | Chemical formula | Relative peak area (%) | CAS |
| --- | --- | --- | --- | --- |
| D1 | α-Pinene | C10H16 | 1.37 | 80-56-8 |
| D2 | β-Phellandrene | C10H16 | 4.28 | 555-10-2 |
| D3 | α-Phellandrene | C10H16 | 0.11 | 99-83-2 |
| D4 | D-limonene | C10H16 | 0.46 | 5989-27-5 |
| D5 | Eucalyptol | C10H18O | 17.27 | 470-82-6 |
| D6 | γ-Terpinene | C10H16 | 0.30 | 99-85-4 |
| D7 | trans-Linalool oxide | C10H18O2 | 0.62 | 34995-77-2 |
| D8 | Linalool | C10H18O | 15.48 | 78-70-6 |
| D9 | β-Terpineol | C10H18O | 0.32 | 138-87-4 |
| D10 | Camphor | C10H16O | 14.57 | 76-22-2 |
| D11 | Borneol | C10H18O | 0.54 | 507-70-0 |
| D12 | 4-Terpineol | C10H18O | 1.08 | 562-74-3 |
| D13 | α-Terpineol | C10H18O | 4.28 | 98-55-5 |
| D14 | trans-Geraniol | C10H18O | 0.15 | 106-24-1 |
| D15 | Bornyl acetate | C12H20O2 | 0.16 | 76-49-3 |
| D16 | δ-Elemene | C15H24 | 0.49 | 20307-84-0 |
| D17 | Methyl eugenol | C10H12O2 | 0.24 | 97-53-0 |
| D18 | β-Caryophyllene | C15H24 | 3.34 | 87-44-5 |
| D19 | γ-Elemene | C15H24 | 0.11 | 29873-99-2 |
| D20 | Humulene | C15H24 | 1.75 | 6753-98-6 |
| D21 | Valencene | C15H24 | 0.26 | 4630-07-3 |
| D22 | γ-Muurolene | C15H24 | 0.23 | 30021-74-0 |
| D23 | Germacrene D | C15H24 | 1.19 | 23986-74-5 |
| D24 | δ-Selinene | C15H24 | 1.00 | 473-14-3 |
| D25 | α-Guaiene | C15H24 | 1.02 | 3691-12-1 |
| D26 | Methyl isoeugenol | C10H12O2 | 1.97 | 97-54-1 |
| D27 | Copaene | C15H24 | 0.41 | 3856-25-5 |
| D28 | α-Muurolene | C15H24 | 0.29 | 10208-80-7 |
| D29 | Elemol | C15H26O | 0.33 | 639-99-6 |
| D30 | trans-Nerolidol | C15H26O | 15.89 | 40716-66-3 |
| D31 | Germacrene B | C15H24 | 0.57 | 15423-57-1 |
| D32 | Spathulenol | C15H24O | 0.66 | 6750-60-3 |
| D33 | caryophyllene oxide | C15H24O | 1.03 | 1139-30-6 |
| D34 | Guaiol | C15H26O | 0.27 | 489-86-1 |
| D35 | Selina-6-en-4-ol | C15H26O | 6.36 |  |
| D36 | Viridiflorol | C15H26O | 0.11 | 552-02-3 |
| D37 | τ-Muurolol | C15H26O | 0.39 | 19912-62-0 |
| D38 | α-Cadinol | C15H26O | 0.21 | 481-34-5 |
| D39 | Juniper camphor | C15H26O | 0.59 | 473-04-1 |
| D40 | Bulnesol | C15H26O | 0.32 | 22451-73-6 |

Table S8. Hub genes and target proteins of *Staphylococcus haemolyticus* treated with *Ginkgo* *biloba* *exocarp* extract.

| KEGG first categories | KEGG second categories | Hub gene | Degree | PDB ID |
| --- | --- | --- | --- | --- |
| Metabolism | Amino acid metabolism | AV904_RS02445 | 8 | 1EJJ |
|  |  | AV904_RS10015 | 8 | 2IQQ |
|  |  | AV904_RS11275 | 8 | 2WE4 |
|  | Carbohydrate metabolism | AV904_RS06675 | 10 | 3P3V |
|  |  | AV904_RS03745 | 8 | 1DTW |
|  |  | AV904_RS00735 | 6 | 2P67 |
|  |  | AV904_RS01405 | 6 | 7EI3 |
|  | Metabolism of cofactors and vitamins | AV904_RS01460 | 10 | 1T0T |
|  |  | AV904_RS07855 | 10 | 1NP3 |
|  |  | AV904_RS11410 | 10 | 1GEE |
|  |  | AV904_RS08275 | 8 | 1N3I |
|  | Energy metabolism | AV904_RS02445 | 6 | 1EJJ |
|  |  | AV904_RS04585 | 6 | 1B0P |
|  | Nucleotide metabolism | AV904_RS01320 | 6 | 1ZM7 |
|  |  | AV904_RS08640 | 6 | 2OSB |
|  | Lipid metabolism | AV904_RS06680 | 4 | 1IUQ |
|  | Global and overview maps | AV904_RS07780 | 4 | 2NWH |
| Genetic Information Processing | Translation | AV904_RS08705 | 22 | 3BBX-L29 |
|  |  | AV904_RS08745 | 22 | 3BBX-L3 |
|  |  | AV904_RS08625 | 20 | 4V4H |
|  | Replication and repair | AV904_RS00245 | 4 | 5XGT |
| Environmental Information Proce | Membrane transport | AV904_RS10985 | 2 | 1Z47 |
|  |  | AV904_RS03785 | 2 | 5NUP |
|  | Signal transduction | AV904_RS01855 | 4 | 3QF4 |
|  |  | AV904_RS03855 | 4 | 6A2J |
| Human Diseases | Drug resistance: antimicrobial | AV904_RS01845 | 2 | 1XHF |

Table S9. Components in *Ginkgo* *biloba* *exocarp* extract.

| Number | Substances | Chemical formula | Relative peak area (%) | CAS |
| --- | --- | --- | --- | --- |
| E1 | ginkgotoxin | C9H13NO3 | 0.42 | 1464-33-1 |
| E2 | sequojaflavone | C31H20O10 | 4.21 | 21763-71-3 |
| E3 | amentoflavone | C30H18O10 | 0.02 | 1617-53-4 |
| E4 | isoginkgetin | C32H22O10 | 9.44 | 548-19-6 |
| E5 | wilsonol A | C13H24O4 | 0.03 | 1446691-57-1 |
| E6 | sciadopitysin | C33H24O10 | 12.07 | 521-34-6 |
| E7 | 6-tetradecylsalicyclic acid | C21H34O3 | 0.06 | 59204-69-2 |
| E8 | 6-tridecylresorcyclic acid | C20H32O4 | 0.02 | 62071-09-4 |
| E9 | Ginkgolic acid 13:0 (6-tridecylsalicyclic acid) | C20H32O3 | 0.31 | 20261-38-5 |
| E10 | 5-tetradecylresorcinol | C20H34O2 | 2.65 | 80368-57-6 |
| E11 | alkly coumarin 15 | C24H36O3 | 0.90 | 60918-92-5 |
| E12 | ɑ-carotene | C40H56 | 0.01 | 432-70-2 |
| E13 | urushiol 15:1 | C21H34O2 | 0.08 | 35237-02-6 |
| E14 | alkly coumarin 13 | C22H32O3 | 39.38 | 94343-84-7 |
| E15 | α-hydroxycardanols 15:0 | C21H36O2 | 18.87 | 444994-98-3 |
| E16 | Ginkgolic acid 13:0 (6-pentadecylsalicyclic acid) | C22H36O3 | 1.42 | 16611-84-0 |
| E17 | a-hydroxycardanols 17:1 | C23H40O2 | 5.33 | 5862-27-1 |
| E18 | ɤ-carotene | C39H56 | 0.03 | 472-93-5 |
| E19 | 6-[8-heptadecenyl] salicyclic acid | C24H38O3 | 4.28 | 69506-63-4 |
| E20 | urushiol 17:1 | C23H38O2 | 0.18 | 54954-20-0 |
| E21 | Ginkgol | C21H34O | 0.30 | 501-26-8 |

Table S10. The detected conditions of GC-MS.

| Instrument | Parameter | Condition |
| --- | --- | --- |
| Chromatographic conditions | Chromatographic column | Agilent J&W DB-5 (30 m×30 m ×0.25 μm) |
|  | Injection volume | 1 μl |
|  | Injection port | 250℃ |
|  | Gas | He |
|  | Temperature program | 40℃ for 5min, 40-150℃ in 10℃/min for 2 min, 150-270 in 5℃/min for 2 min |
| Mass spectrometry conditions | Ionization | EI |
|  | Ionization energy | 70 eV |
|  | Ion power temperature | 300℃ |
|  | Interface temperature | 250℃ |
|  | Solvent delay | 2.5 min |

Table S11. The detected conditions of HPLC-Qe.

| Instrument | Parameter | Condition |
| --- | --- | --- |
| Chromatographic conditions | Chromatographic column | ACQUITY UPLC BEH C18 (1.7μm, 2.1×100 mm) |
|  | Precolumn | VanGuardTM BEH C18 1.7μm |
|  | Column temperature | 40℃ |
|  | Injection volume | 5 μL |
|  | Velocity | 0.25 mL/min |
|  | Mobile phase | A (MeOH), B (0.1% formic acid-water), gradient elute |
|  | Gradient elution program | 20% A and 80% B in 3 min, 100% A in 35 min, 100% A in 45 min, 20% A and 80% B in 50 min |
| Mass spectrometry conditions | Ionization | ESI, positive and negative ion modes |
|  | Gas | N2 |
|  | Runtime(min) | 0-32 |
|  | Scan type | Full MS dd-MS2 |
|  | Scan range | 50-750 |
|  | Spray Voltage(\|kV\|) | 3.5(+), 3.0(-) |
|  | Chrom.peak width (FWHM, s) | 15 |
|  | Resolution | 70,000 (MS), 17,500 (MS2) |
|  | AGC target | 3e6 (MS), 1e5 (MS2) |
|  | Maximum IT (ms) | 100 (MS);50 (MS2) |
|  | Sheat gas | 30 |
|  | Aux gas flow | 5 |
|  | Sweep gas | 2 |
|  | Capillary temp (℃) | 300 |
|  | S-lens RF level | 50 |
|  | Aux gas heater temp (℃) | 250 |
|  | TopN | 8 |
|  | NCE | 10, 20, 40 |

Table S12. Binding free energy calculation procedure and corresponded command.

| Procedure | Commands |
| --- | --- |
| Extracting trace file of protein-compound complex | vmd -dispdev text -psf "ionized.psf" -e stripDCD.vmd -args protein,or,resname,LIG "ionized.dcd" "complex" ionized.psf ionized.pdb |
| MDS of complex | namd2 complex.namd > complex.log |
| Extract protein trajectory file | vmd -dispdev text -psf "ionized.psf" -e stripDCD.vmd -args protein "ionized.dcd" "protein" ionized.psf ionized.pdb |
| MDS of protein | namd2 protein.namd > protein.log |
| Extract compound trajectory file | vmd -dispdev text -psf "ionized.psf" -e stripDCD.vmd -args resname,LIG "ionized.dcd" "ligand" ionized.psf ionized.pdb |
| MDS of compound | namd2 ligand.namd > ligand.log |
| Binding free energy calculation | molaical.exe -mmgbsa -c complex.log -r protein.log -l ligand.log |

Table S13. Other antimicrobial activity of screened components in essential oil from *Cinnamomum* *camphora* in previous researches.^[14–21]^

| Compounds | Antimicrobial activity |
| --- | --- |
| D2 | **Antibacterial activity**: against *S*. *mutans* and *S*. *sobrinus* with 8 mm inhibition diameter |
| D3 | **Antibacterial activity**: against *S*. *mutans* and *S*. *sobrinus* with 8 mm inhibition diameter |
| D16 | **Antitumoral activity**: 50% development inhibition of nude mice tumors |
| D19 | **Insecticidal activity**: inhibit *Leishmania* growth with IC_50_ of 9.82 μg/mL |
| D24 | **Insecticidal activity**: contact toxicity against *D*. *melanogaster* adults with LD_50_ of 0.55 µg/adult |
| D29 | **Insecticidal activity**: contact toxicity against *L*. *entomophila* and adults *T*. *castaneum* with LD_50_ of 35.19 µg/cm^2^ and 8.89 µg/adult  **Antifungal activity**: against *L*. *Sulphureus* and *L*. *Betulina* with IC_50_ of 30.5 and 40.5 μg/mL |
| D32 | **Insecticidal activity**: against *Metopolophium dirhodum* with LD_50_ of 4.3 mL/mL  **Antibacterial activity**: against *S*. *aureus* with MIC of 136 μg/mL |
| D40 | **Insecticidal activity**: induce 15% *R*. *microplus* larval mortality  **Antifungal activity**: against *L*. *Sulphureus* and *L*. *Betulina* with IC_50_ of 23.1 and 60.2 μg/mL |

Table S14. Other bioactivity of screened components in *Ginkgo* *biloba* *exocarp* extract in previous studies^[22–34]^.

| Compounds | Bioactivity |
| --- | --- |
| E2 | **Antiviral activity**: inhibit Dengue 2 NS5 RNA-dependent RNA polymerase with IC_50_ of 0.25 μg/mL  **Anticancer activity**: inhibit MCF-7 cells with IC_50_ of 23 μg/mL  **Anti-Alzheimer's disease**: inhibit Aβ fibrils formation with IC_50_ of 0.16 μg/mL |
| E5 | **Antiviral activity**: inhibit Dengue 2 NS5 RNA-dependent RNA polymerase with IC_50_ of 0.00025 mg/mL  **Antioxidant activity**: scavenge DPPH and 1O_2_ radical with IC_50_ of 25 and 3.0 μg/mL |
| E8 | **Antibacterial activity**: against *S*. *aureus* with MIC of 2.1 μg/mL |
| E9 | **Anti-diabetes**: inhibit Glycerol-3-phosphate dehydrogenase with IC_50_ of 3.0 μg/ml  **Anticancer activity**: inhibit U251 and SMMC7721 cells with IC_50_ of 35.7 and 32.2 μg/mL |
| E12 | **Antitumoral activity**: decrease 63.8% incidence of tumor-bearing mice  **Anti-inflammatory activity**: decrease value of IL-1β, IL-6, MPO, TGF-β1 and TNF-α  **Antioxidant activity**: increase value of CAT, GSH, decrease value of MDA, SOD, NO |
| E13 | **Antioxidant activity**: inhibit soybean and potato lipoxygenases with IC_50_ of 3.2 and 4.6 μg/mL  **Anti-renal fibrosis**: hinder 32.1% renal fibrosis with concentration of 40 μg/mL |
| E17 | **Antibacterial activity**: against *S*. *aureus* with MIC > 800 μg/mL |
| E18 | **Anti-renal fibrosis**: hinder hepatic fibrosis with IC_50_ of 107 μg/mL |
| E19 | **Anti-diabetes**: inhibit Glycerol-3-phosphate dehydrogenase with IC_50_ of 0.9 μg/ml |
| E21 | **Anticancer activity**: inhibit A549, U251 and SMMC7721 cells with IC_50_ of 11.7, 10.6 and 8.5 μg/mL |

Table S15. The time and cost comparation of TCMD and traditional methods.^a)^

| Parameters | TCMD | Traditional methods |
| --- | --- | --- |
| Time | Additional preparation and transcriptome analysis:  1-2 days | Chemical purchase:  1~ days |
|  | Molecular docking:  10-20 mins | Plate inhibition experiment:  10~ days |
|  | Result analysis:  ~10 mins | Result statistics:  1~ days |
| Cost | Molecular docking (Online supercomputing platform):  550 $ | Chemical purchase:  4,000 to 5,500 $  (14-280 $ per chemical) |

^a)^(Antimicrobial mixture containing 100 compounds)

**Data S1. (separate file)** GC-MS and HPLC-Qe results for **Figure 2a**.

**Data S2. (separate file)** Docking scores of molecular docking for HTL-AP.

**Data S3. (separate file)** Docking scores of molecular docking for **e**ssential oil from *Cinnamomum* *camphora*.

**Data S4. (separate file)** Docking scores of molecular docking for *Ginkgo* *biloba* *exocarp* extract.

**Data S5. (separate file)** Different Expressed Genes for **Figure S3a**.

**Data S6. (separate file)** KEGG Function Annotation Analysis of DEGs for **Figure S3b**.

**Data S7. (separate file)** KEGG Function Enrichment Analysis of DEGs for **Figure S3c**.

**Software S1. (separate file)** Scripts for molecular dynamic simulation by NAMD.

**Software S2. (separate file)** Scripts for binding free energy calculation by MolAICal.

References

[1] S. Das, P. K. Meher, A. Rai, L. M. Bhar, B. N. Mandal, *PLoS One* **2017**, *12*, 1.

[2] C. Wurzenberger, D. W. Gerlich, *Nat. Rev. Mol. Cell Biol.* **2011**, *12*, 469.

[3] J. Kobayashi, Y. Matsuura, *Protein Sci.* **2017**, *26*, 2105.

[4] X. Li, M. Liu, T. Huang, K. Yang, S. Zhou, Y. Li, J. Tian, *Postharvest Biol. Technol.* **2021**, *171*, 111343.

[5] H. G. Enoch, A. Catala, P. Strittmatter, *J. Biol. Chem.* **1976**, *251*, 5095.

[6] H. Wang, M. G. Klein, H. Zou, W. Lane, G. Snell, I. Levin, K. Li, B. C. Sang, *Nat. Struct. Mol. Biol.* **2015**, *22*, 581.

[7] Z. Xia, F. S. Mathews, *J. Mol. Biol.* **1990**, *212*, 837.

[8] A. K. Padyana, S. Gross, L. Jin, G. Cianchetta, R. Narayanaswamy, F. Wang, R. Wang, C. Fang, X. Lv, S. A. Biller, L. Dang, C. E. Mahoney, N. Nagaraja, D. Pirman, Z. Sui, J. Popovici-Muller, G. A. Smolen, *Nat. Commun.* **2019**, *10*, 97.

[9] X. Huang, G. Zhang, T. Tang, X. Gao, T. Liang, *Biochim. Biophys. Acta - Rev. Cancer* **2022**, *1877*, 188696.

[10] J. Mu, J. Zhou, Q. Gong, Q. Xu, *Comput. Struct. Biotechnol. J.* **2022**, *20*, 368.

[11] V. Gomez, A. Hergovich, *Cell-Cycle Control and DNA-Damage Signaling in Mammals*, Vol. 1, Elsevier Inc. **2016**.

[12] H. N. Ki, S. J. Kim, M. Y. Kim, H. K. Jae, Y. S. Yeo, C. M. Lee, H. K. Jun, Y. H. Kwang, *Proteins Struct. Funct. Genet.* **2008**, *73*, 788.

[13] K. H. Nam, M. W. Sung, K. Y. Hwang, *Biochem. Biophys. Res. Commun.* **2010**, *391*, 1131.

[14] O. Choi, S. K. Cho, J. Kim, C. G. Park, J. Kim, *Asian Pac. J. Trop. Biomed.* **2016**, *6*, 308.

[15] Y.-Q. Yao, X. Ding, Y.-C. Jia, C.-X. Huang, Y.-Z. Wang, Y.-H. Xu, *Cancer Lett.* **2008**, *264*, 127.

[16] T. A. de Lima Nunes, L. H. Costa, J. M. S. de Sousa, V. M. R. de Souza, R. R. L. Rodrigues, M. da Conceição Albuquerque Val, A. C. T. da Cunha Pereira, G. P. Ferreira, M. V. da Silva, J. M. Da Costa, L. M. C. Véras, R. C. Diniz, K. A. F. Rodrigues, *Chem. Biol. Interact.* **2021**, 109429.

[17] S. S. Chu, G. H. Jiang, Z. L. Liu, *Pest Manag. Sci.* **2011**, *67*, 1253.

[18] C. C. Wu, S. L. Huang, C. H. Ko, H. T. Chang, *Molecules* **2022**, *27*, 1.

[19] X. Pan, H. Xiao, X. Hu, Z. L. Liu, *Z. Naturforsch. C.* **2023**, *78*, 83.

[20] A. Ulubelen, G. Topcu, C. Eri§, U. Sönmez, M. Kartal, S. Kurucu, C. Bozok-Johansson, *Phytochemistry* **1994**, *36*, 971.

[21] D. A. R. Luns, R. Martins, S. Pombal, J. M. L. Rodilla, N. W. Githaka, I. da Silva Vaz, C. Logullo, *Exp. Appl. Acarol.* **2021**, *83*, 609.

[22] K. S. Nagabhushana, S. Umamaheshwari, F. E. Tocoli, S. K. Prabhu, I. R. Green, C. S. Ramadoss, *J. Enzyme Inhib. Med. Chem.* **2002**, *17*, 255.

[23] Y. Xie, X. Zhou, J. Li, X. chang Yao, W. li Liu, P. sheng Xu, G. shan Tan, *Bioorganic Med. Chem. Lett.* **2022**, *56*, 128486.

[24] J. Irie, M. Murata, S. Homma, *Biosci. Biotechnol. Biochem.* **1996**, *60*, 240.

[25] V. P. Bagla, L. J. McGaw, E. E. Elgorashi, J. N. Eloff, *BMC Complement. Altern. Med.* **2014**, *14*, 2.

[26] P. Coulerie, M. Nour, A. Maciuk, C. Eydoux, J. C. Guillemot, N. Lebouvier, E. Hnawia, K. Leblanc, G. Lewin, B. Canard, B. Figadère, *Planta Med.* **2013**, *79*, 1313.

[27] E. Y. Choi, S. S. Kang, S. K. Lee, B. H. Han, *Biomol. Ther.* **2020**, *28*, 145.

[28] I. Kubo, K. I. Nihei, K. Tsujimoto, *J. Agric. Food Chem.* **2003**, *51*, 7624.

[29] Y. E. Lee, T. Kodama, H. Morita, *J. Nat. Med.* **2023**, *77*, 298.

[30] G. Zhang, Y. Liu, P. Liu, *J. Agric. Food Chem.* **2018**, *66*, 12257.

[31] X. M. Yang, Y. F. Wang, Y. Y. Li, H. Le Ma, *Fitoterapia* **2014**, *98*, 66.

[32] Q. Gu, Y. Li, Y. Chen, P. Yao, T. Ou, *Sciadopitysin: Active component from Taxus chinensis for anti-Alzheimer’s disease*, Vol. 27, Taylor & Francis **2013**.

[33] J. Ai, J. Nie, J. He, Q. Guo, M. Li, Y. Lei, Y. Liu, Z. Zhou, F. Zhu, M. Liang, Y. Cheng, F. F. Hou, *J. Am. Soc. Nephrol.* **2015**, *26*, 1827.

[34] L. Hou, Y. Li, Q. Wu, M. Li, E. A. Older, X. Tang, P. Nagarkatti, M. Nagarkatti, Y. Liu, L. Li, D. Fan, T. S. Bugni, Z. Shang, J. Li, *Bioorg. Chem.* **2021**, *112*, 104925.
